# Effect of Maternal Egg Intake During the Early Neonatal Period and Risk of Infant Egg Allergy at 12 Months Among Breastfeeding Mothers: A Randomized Clinical Trial

**DOI:** 10.1001/jamanetworkopen.2023.22318

**Published:** 2023-07-10

**Authors:** Ken-ichi Nagakura, Sakura Sato, Wakako Shinahara, Hiroshi Kido, Hidetoshi Fujita, Takanori Yanai, Nao Akiyama, Masaki Futamura, Hiroshi Koga, Michimasa Fujiwara, Hideo Kaneko, Hiroaki Taniguchi, Eishi Makita, Kyohei Takahashi, Noriyuki Yanagida, Motohiro Ebisawa, Mitsuyoshi Urashima

**Affiliations:** 1 Department of Pediatrics, National Hospital Organization Sagamihara National Hospital, Kanagawa, Japan; 2 Department of Pediatrics, Jikei University School of Medicine, Tokyo, Japan; 3 Department of Allergy, Clinical Research Center for Allergy and Rheumatology, National Hospital Organization Sagamihara National Hospital, Kanagawa, Japan; 4 Division of Enzyme Chemistry, Institute for Enzyme Research, Tokushima University, Tokushima, Japan; 5 Department of Pediatrics, Aiwa hospital, Saitama, Japan; 6 Department of Pediatrics, National Hospital Organization Yokohama Medical Center, Kanagawa, Japan; 7 Department of Pediatrics, National Hospital Organization Saitama Hospital, Saitama, Japan; 8 Department of Pediatrics, National Hospital Organization Nagoya Medical Center, Aichi, Japan; 9 Department of Pediatrics, National Hospital Organization Beppu Medical Center, Oita, Japan; 10 Department of Pediatrics, National Hospital Organization Fukuyama Medical Center, Hiroshima, Japan; 11 Department of Pediatrics, National Hospital Organization Nagara Medical Center, Gifu, Japan; 12 Department of Pediatric Medical Care, Gifu Prefectural General Medical Center, Gifu, Japan; 13 Department of Pediatrics, Konan Medical Center, Hyogo, Japan; 14 Department of Pediatrics, Saitama Medical Center Jichi Medical University, Saitama, Japan; 15 Division of Molecular Epidemiology, Jikei University School of Medicine, Tokyo, Japan

## Abstract

**Importance:**

Egg introduction in infants at age 4 to 6 months is associated with a lower risk of immunoglobulin E–mediated egg allergy (EA). However, whether their risk of EA at age 12 months is affected by maternal intake of eggs at birth is unknown.

**Objective:**

To determine the effect of maternal egg intake during the early neonatal period (0-5 days) on the development of EA in breastfed infants at age 12 months.

**Design, Setting, and Participants:**

This multicenter, single-blind (outcome data evaluators), randomized clinical trial was conducted from December 18, 2017, to May 31, 2021, at 10 medical facilities in Japan. Newborns with at least 1 of 2 parents having an allergic disease were included. Neonates whose mothers had EA or were unable to consume breast milk after the age of 2 days were excluded. Data were analyzed on an intention-to-treat basis.

**Interventions:**

Newborns were randomized (1:1) to a maternal egg consumption (MEC) group, wherein the mothers consumed 1 whole egg per day during the first 5 days of the neonate’s life, and a maternal egg elimination (MEE) group, wherein the mothers eliminated eggs from their diet during the same period.

**Main Outcomes and Measures:**

The primary outcome was EA at age 12 months. Egg allergy was defined as sensitization to egg white or ovomucoid plus a positive test result in an oral food challenge or an episode of obvious immediate symptoms after egg ingestion.

**Results:**

Of the 380 newborns included (198 [52.1%] female), 367 (MEC: n = 183; MEE: n = 184) were followed up for 12 months. On days 3 and 4 after delivery, the proportions of neonates with ovalbumin and ovomucoid detection in breast milk were higher in the MEC group than in the MEE group (ovalbumin: 10.7% vs 2.0%; risk ratio [RR], 5.23; 95% CI, 1.56-17.56; ovomucoid: 11.3% vs 2.0%; RR, 5.55; 95% CI, 1.66-18.55). At age 12 months, the MEC and MEE groups did not differ significantly in EA (9.3% vs 7.6%; RR, 1.22; 95% CI, 0.62-2.40) or sensitization to egg white (62.8% vs 58.7%; RR, 1.07; 95% CI, 0.91-1.26). No adverse effects were reported.

**Conclusions and Relevance:**

In this randomized clinical trial, EA development and sensitization to eggs were unaffected by MEC during the early neonatal period.

**Trial Registration:**

UMIN Clinical Trials Registry: UMIN000027593

## Key Points

**Question:**

Does maternal intake of hen’s eggs at birth affect the risk of immunoglobulin E–mediated egg allergy in infants aged 12 months?

**Findings:**

In this randomized clinical trial involving 380 breastfed infants whose parents had an allergic disease, maternal consumption of 1 whole egg per day during the first 5 days after delivery did not affect egg allergy development and sensitization to egg white in the infants compared with complete egg avoidance by the mothers during the same period. No adverse effects were observed.

**Meaning:**

The findings of this randomized clinical trial indicate that egg allergy development is unaffected by maternal egg consumption during the very early neonatal period.

## Teaser

This randomized clinical trial examines the development of egg allergy and sensitization in breastfed infants whose mothers ate eggs in the first 5 days after delivery.

## Introduction

The prevalence of food allergy is increasing,^1,2,3,4,5^ estimated at approximately 10% in children.^4,5,6,7^ Notably, the hen’s egg is one of the most common causative foods for food allergies and anaphylaxis.^8,9,10,11^ Thus, preventing egg allergy (EA) is important for children.

Oral tolerance induction to prevent EA and oral immunotherapy for patients with EA has shown promising results.^12,13,14,15,16,17,18,19,20^ For example, in several oral immunotherapy trials, although high-dose egg protein ingestion induced allergic symptoms, low-dose egg protein ingestion could induce tolerance with fewer allergic symptoms.^17,18,19,20,21,22^ In addition, although several randomized clinical trials (RCTs) and a meta-analysis reported that early consumption of eggs from age 3 to 6 months prevented EA,^14,15,16,23^ a certain number of infants had already developed EA at the time of enrollment.^14,16,24,25,26,27^ A case series study showed that EA had already developed by age 3 months.^28^ Based on these results, intervention earlier than age 3 to 6 months would be desirable for the primary prevention of EA.^5^

In 2019, the ABC (Atopy Induced by Breastfeeding or Cow’s Milk Formula) trial showed that consuming cow’s milk formula protein during the first 3 days of life increased the risk of milk allergy.^29^ Additionally, 2 cohort trials found that milk protein ingestion for 1 or 3 days after birth was associated with an increased risk of milk allergy.^30,31^ Generally, egg proteins secreted into breast milk as a result of the maternal egg intake are minuscule.^32,33,34^ Therefore, we hypothesized that administering eggs via breastfeeding during the early neonatal period (0-5 days) prevents EA, acting like spontaneous low-dose oral immunotherapy. To examine this hypothesis, this multicenter RCT was conducted to assess whether maternal egg consumption (MEC) or elimination by mothers in the first 5 days after delivery prevents infants’ EA.

## Methods

### Study Design and Ethics Approval

This multicenter, single-blind (outcome data evaluators) RCT was conducted from December 18, 2017, to May 31, 2021, at 10 facilities in Japan. The trial protocol is provided in Supplement 1. This study was conducted in accordance with the regulatory requirements and approved by the Central Ethical Review Committee for Clinical Research of the National Hospital Organization. Informed consent was obtained from the mother of each infant before participation in this study; financial compensation was not provided. Participants’ clinical data were managed using anonymized research identification. We followed the Consolidated Standards of Reporting Trials (CONSORT) reporting guideline. Data were analyzed on an intention-to-treat basis.

### Participants

Inclusion criteria were neonates with at least 1 of the 2 parents having a current medically diagnosed allergic disease (at least 1 of the following: atopic dermatitis, bronchial asthma, food allergy, allergic rhinitis, and allergic conjunctivitis) as determined by a physician, which was considered a high risk factor for developing food allergy.^7,35^ Exclusion criteria were as follows: (1) neonates younger than 37 weeks of gestational age, (2) neonates with a birth weight less than 2300 g, (3) neonates with birth asphyxia (Apgar score <3 points at 5 minutes), (4) neonates who needed to be admitted to the newborn intensive care unit, (5) neonates who were unable to tolerate breast milk at all after the age of 2 days without specifying whether the route was by breast or bottle, and (6) neonates whose mothers were diagnosed with EA by a physician and could not consume eggs.

### Randomization and Grouping

Allocation was performed anonymously using the envelope method, and each facility randomized allocation adjustment factors using the substitution block method. Physicians who followed up the children and evaluated their food allergies were blinded to the randomization results.

Mothers and their neonates were randomized (1:1 ratio) into an MEC group, wherein the mother consumed 1 whole egg per day between 0 and 5 days after delivery, and a maternal egg elimination (MEE) group, wherein the mother eliminated eggs from her diet between 0 and 5 days after delivery.

### Treatment Procedures

The eggs consumed by mothers in the MEC group were boiled whole eggs prepared by the nutrition department of each medical facility. The mothers in the MEC group consumed 1 cooked egg per day in addition to their normal breakfast from days 0 to 5, whereas those in the MEE group received a hospital diet that eliminated eggs from days 0 to 5. In Japan, the normal postpartum hospital stay is 5 days. No dietary restrictions were imposed on either group after discharge. After 1 month, both groups were instructed to perform skin care, and aggressive topical corticosteroid treatment was administered for eczema. The treatment procedures and measurements are shown in eFigure 1 in Supplement 2. At age 4 and 12 months, the children’s blood was assessed for egg sensitization. If the egg white– or ovomucoid (OVM)-specific immunoglobulin (Ig)E (sIgE) level was 0.1 allergen-specific kilo units per liter (kU_A_/L) or more, an oral food challenge (OFC) was performed to assess EA. For the OFC, the total challenge dose was administered in a stepwise manner (250, 775, 3100, and 6200 mg of egg protein) based on the Japanese guidelines for food allergy.^7,36,37,38^ A positive OFC result was defined by a blinded physician as the appearance of objective or prolonged subjective symptoms. Details of the OFC methods have been previously described.^22,36,37,38,39^

### Outcomes and Measures

The primary outcome was EA at 12 months, defined as both a positive sensitization to egg white or OVM sIgE greater than or equal to 0.1 kU_A_/L and a positive test result of OFC or an episode of obvious immediate symptoms after egg ingestion. When the infants were aged 12 months, blinded physicians evaluated the presence or absence of EA. The secondary outcomes were egg protein concentrations in breast milk collected on days 3 to 4 and at age 1 month; sensitization to egg, milk, and wheat; food protein–induced enterocolitis syndrome due to egg, milk, and wheat; and eczema.

Blood tests were performed at age 4 and 12 months to measure total IgE and egg white, OVM, cow’s milk, casein, wheat, and ω-5 gliadin sIgE (ImmunoCAP; Thermo Fisher Scientific/Phadia AB), and an sIgE antibody titer of 0.10 kU_A_/L or higher was considered sensitization. To measure ovalbumin (OVA) and OVM content in breast milk, on the third or fourth day after delivery, 1 mL of breast milk was collected 3 times in total (1, 3, and 6 hours after breakfast). Concentrations of OVA and OVM in breast milk were determined at the University of Tokushima, using a fluorescently labeled secondary antibody after the anti-OVA or OVM rabbit polyclonal IgG antibody was loaded on the chip and reacted with breast milk stock solution.^40,41^ The detection sensitivity of OVA was greater than or equal to 0.20 ng/mL and the detection sensitivity of OVM was greater than or equal to 0.78 ng/mL Details of the measurement method are described in the eMethods in Supplement 2.

### Statistical Analysis

Infants who were not evaluated for EA at 12 months were excluded. Participants who did not adhere to the intervention were evaluated based on their allocation group. Comparisons between groups were performed using the Fisher exact test for categorical variables and the Wilcoxon rank sum test for continuous variables. Data requiring a distribution of normality were not analyzed. Effects of intervention, such as maternal intake of eggs at birth, were calculated using RRs with 95% CIs, using the Fisher exact test for incidence of food allergy, eczema, and sensitization.

Statistical significance was set at *P* < .05. All data were processed and summarized using SPSS, version 25.0 (IBM Corp).

#### Sample Size

Since only neonates at a high risk of developing food allergies were included in this study,^35^ the EA at 12 months in the MEE group was assumed to be 15%. The preventive effect of early intervention was assumed to be 67%, and that of EA in the MEC group was assumed to be 5%. At a power level of 0.8, assuming loss to follow-up of 20% and considering the differences between centers for conducting this study at 10 facilities throughout Japan, the total number of participants required was 380.

#### Post Hoc Analysis

The characteristics of infants who developed (EA group) or did not develop (non-EA group) EA were assessed. Clinical factors associated with the development of EA, such as atopic disease, eczema, and intervention of egg ingestion, were analyzed. The risk ratios (RRs) with 95% CIs comparing the factors influencing EA development are reported based on a logistic regression analysis.

## Results

### Participants

**Figure 1.**
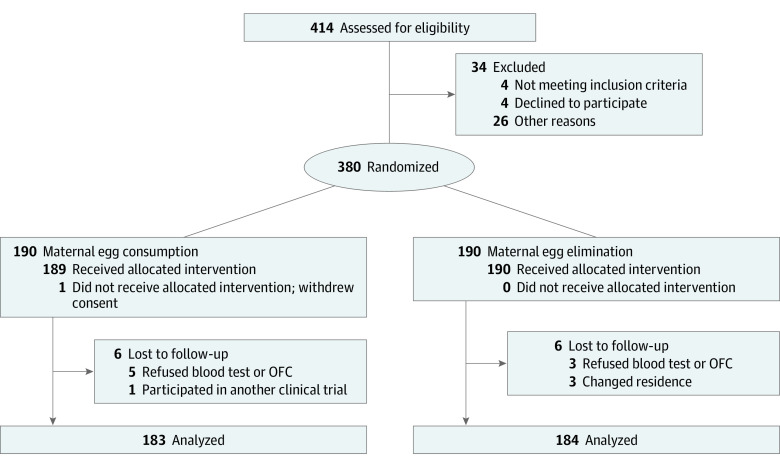
Participant Recruitment Flow OFC indicates oral food challenge.

Of 414 included infants, 34 were excluded according to the inclusion and exclusion criteria, and the remaining 380 were enrolled in the RCT (198 females [52.1%] and 182 males [47.9%]): 190 in the MEC group and 190 in the MEE group. Additionally, 13 voluntarily withdrew or were lost to follow-up. A total of 183 infants (96.3%) in the MEC group and 184 infants (96.8%) in the MEE group completed follow-up at age 12 months and were included in the analysis. The follow-up rate for all infants was 96.6% (Figure 1). Table 1 reports the baseline characteristics of all enrolled participants.

**Table 1.** Baseline Characteristics of Participants in the MEC and MEE Groups

Characteristic	No. (%)	
	MEC group (n = 190)	MEE group (n = 190)
Sex		
Male	90 (47.4)	92 (48.4)
Female	100 (52.6)	98 (51.6)
Gestational age, median (IQR), wk	39 (38-40)	39 (38-40)
Body weight, median (IQR), g	3085 (2823.5-3263)	3000 (2820-3275)
Current disease of mother		
Allergic disease	155 (81.6)	157 (82.6)
Food allergy	32 (16.8)	42 (22.1)
Current disease of father		
Allergic disease	156 (82.1)	150 (78.9)
Food allergy	20 (10.5)	19 (10.0)
No. of siblings		
0	120 (63.2)	108 (56.8)
1	47 (24.7)	56 (29.5)
2	21 (11.1)	22 (11.6)
3	1 (0.5)	2 (1.1)
≥4	1 (0.5)	2 (1.1)
Cesarean delivery	42 (22.1)	47 (24.7)

Abbreviations: MEC, maternal egg consumption; MEE, maternal egg elimination.

### Adherence to Intervention and Nutrition Intake Status

During 0 to 5 days of neonatal age, the mothers of 187 neonates (98.4%) in the MEC group ingested a whole egg, whereas the mothers of 184 neonates (96.8%) in the MEE group eliminated eggs from their diets. The adherence rate for all mothers was 97.6%. On day 3, the median amount of breast milk consumed per feed was 12 mL for both groups (eTable 1 in Supplement 2).

At ages 1, 4, 7, and 10 months, the infants’ nutritional methods (ie, exclusively breast milk, mixed feeding, formula intake, and maternal egg intake) did not differ significantly between the MEC and MEE groups (eTable 2 and eTable 3 in Supplement 2).

### Ovalbumin and Ovomucoid Levels in Breast Milk

**Figure 2.**
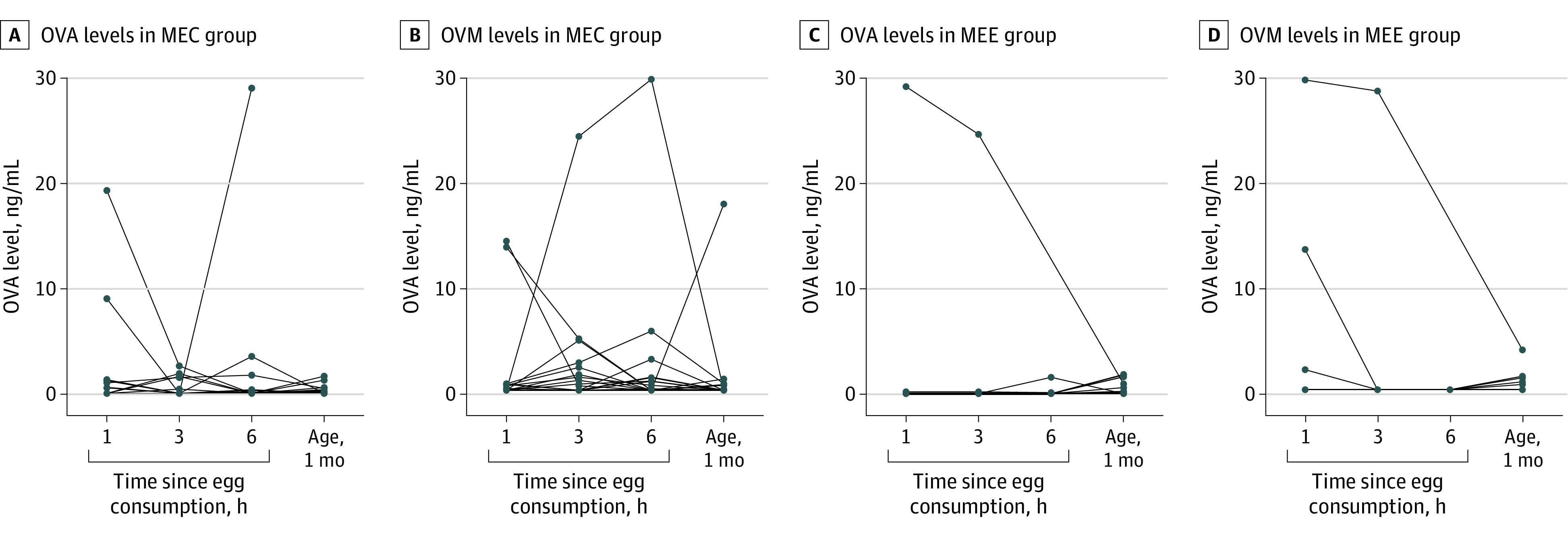
Ovalbumin (OVA) and Ovomucoid (OVM) Levels in Breast Milk After Ingestion of a Whole Egg or During the Elimination of Eggs A, OVA levels in the maternal egg consumption (MEC) group. B, OVM levels in the MEC group. C, OVA levels in the maternal egg elimination (MEE) group. D, OVM levels in the MEE group. The midwife assisted and collected breast milk at a time when the mother was not feeding.

On days 3 to 4, the proportions of neonates with OVA and OVM detected in their mothers’ breast milk were higher in the MEC group than in the MEE group (OVA: 10.7% vs 2.0%; RR, 5.23; 95% CI, 1.56-17.56; OVM: 11.3% vs 2.0%; RR, 5.55; 95% CI, 1.66-18.55) (eTable 4 in Supplement 2). Additionally, peak levels of OVA (*P* = .004) and OVM and OVM in breast milk at 3 and 6 hours after egg consumption were significantly higher in the MEC group than in the MEE group (Figure 2). However, at age 1 month, the OVA and OVM levels in breast milk were not significantly different between the MEC and MEE groups.

### EA and Other Outcomes

The effects of MEC at birth on EA at 12 months of age (primary outcome) and other secondary outcomes are provided in Table 2. Egg allergy was not significantly different between the MEC and MEE groups (9.3% vs 7.6%; RR, 1.22; 95% CI, 0.62-2.40) (Table 2); the overall EA prevalence was 8.4%. Additionally, the eczema, milk, and wheat allergies at age 1 and 4 months did not differ significantly between groups. Food protein–induced enterocolitis syndrome due to eggs was observed in 1 infant in each group. The sensitization to egg white was 30.1% at age 4 months and 62.8% at age 12 months in the MEC group and 26.6% at age 4 months and 58.7% at age 12 months in the MEE group (MEC vs MEE: 4 months: RR, 1.13; 95% CI, 0.81-1.56; 12 months: RR, 1.07; 95% CI, 0.91-1.26). The overall sensitization to egg at 12 months was 60.7%. Moreover, sensitization to milk and wheat did not differ significantly between the 2 groups (Table 2; eFigure 2 in Supplement 2). An interim analysis was performed when the primary outcome was achieved for every 100 infants, and all analyses exceeded the Peto cutoff of 0.001, and this study continued.

**Table 2.** Prevalence of Eczema at Age 1 and 4 Months and Food Allergy and Sensitization at Age 12 Months

Outcome	No. (%)		RR (95% CI)^a^	*P* value^b^
	MEC group (n = 183)	MEE group (n = 184)		
At 1 mo assessment				
Eczema	71 (38.8)	72 (39.1)	0.99 (0.77-1.28)	>.99
At 4 mo assessment				
Eczema	64 (35.0)	67 (36.4)	0.96 (0.73-1.26)	.83
Sensitization to egg	55 (30.1)	49 (26.6)	1.13 (0.81-1.56)	.49
Sensitization to milk	30 (16.4)	34 (18.5)	0.89 (0.57-1.39)	.68
Sensitization to wheat	8 (4.4)	11 (6.0)	0.73 (0.30-1.78)	.64
At 12 mo assessment				
IgE-mediated egg allergy	17 (9.3)	14 (7.6)	1.22 (0.62-2.40)	.58
IgE-mediated milk allergy	3 (1.6)	3 (1.6)	1.01 (0.21-4.92)	>.99
IgE-mediated wheat allergy	2 (1.1)	0	NA	NA
Sensitization to egg	115 (62.8)	108 (58.7)	1.07 (0.91-1.26)	.46
Sensitization to milk	68 (37.2)	73 (39.7)	0.94 (0.72-1.21)	.67
Sensitization to wheat	47 (25.7)	46 (25.0)	1.03 (0.72-1.46)	.91
FPIES due to egg	1 (0.5)	1 (0.5)	1.01 (0.06-15.95)	>.99

Abbreviations: FPIES, food protein–induced enterocolitis syndrome; MEC, maternal egg consumption; MEE, maternal egg elimination; NA, not applicable; RR, risk ratio.

^a^ Estimated as the risk of the effects of intervention (ie, maternal intake of eggs at birth) on outcomes.

^b^ To clarify whether the intervention significantly affected the outcomes, *P* values were analyzed using the Fisher exact test.

### Adverse Events

No adverse events were observed in either group during the intervention period (days 0-5). Regarding serious adverse events during the entire study period up to 12 months, 1 patient in the MEE group was hospitalized because of a urinary tract infection at age 9 months.

### Post Hoc Analysis

Between the EA and non-EA groups, statistical significance was observed for cesarean delivery, OVM detection in breast milk on days 3 and 4, and eczema at 1 month (Table 3). Logistic regression analysis revealed that OVM detection in breast milk on days 3 and 4 (adjusted RR [ARR], 4.04; 95% CI, 1.58-10.32) and eczema at 1 month (ARR, 2.85; 95% CI, 1.39-5.85) were significant risk factors for developing EA (eTable 5 in Supplement 2).

**Table 3.** Comparison of Characteristics Between Children Who Developed Egg Allergy and Those Who Did Not Develop Egg Allergy

Variable	No. (%)		*P* value
	Egg allergy (n = 31)	No egg allergy (n = 336)	
**At birth**			
Sex			
Male	17 (54.8)	157 (46.7)	.40
Female	14 (45.2)	179 (53.3)	
No. of siblings			
0	21 (67.7)	200 (59.5)	.37
1	8 (25.8)	91 (27.1)	
2	1 (3.2)	40 (11.9)	
3	1 (3.2)	2 (0.6)	
≥4	0	3 (0.9)	
Father with allergic diseases	26 (83.9)	274 (81.5)	.75
Father with FA	3 (9.7)	36 (10.7)	.86
Mother with allergic diseases	29 (93.5)	271 (80.7)	.08
Mother with FA	8 (25.8)	61 (18.2)	.30
Cesarean delivery	12 (38.7)	75 (22.3)	.04
During hospitalization			
Intervention of egg ingestion based on RCT	17 (54.8)	166 (49.4)	.56
Formula protein ingestion during days 0-3	16/20 (80.0)	166/212 (78.3)	>.99
OVA detection in breast milk on days 3-4	3/23 (13.0)	16/274 (5.8)	.18
OVM detection in breast milk on days 3-4	4/23 (17.4)	17/274 (6.2)	.03
**At 1 mo**			
Eczema	20 (64.5)	123 (36.6)	.006
Complete breastfeeding	10 (32.3)	104 (31.0)	.83
Frequency of mothers’ egg consumption, median, times/wk (IQR)	5 (5-7)	5 (2-7)	.87
Amount of mothers’ egg consumption, median, No. (IQR)	1 (1-1)	1 (1-1)	.73
OVA detection in breast milk	1/24 (4.2)	17/269 (6.3)	.68
OVM detection in breast milk	0/24	12/269 (4.5)	>.99

Abbreviations: FA, food allergy; OVA, ovalbumin; OVM, ovomucoid; RCT, randomized clinical trial.

In the MEC group, the EA at 12 months was 50.0% in infants who had detectable OVM levels and developed eczema, 13.3% in those who had detectable OVM levels and did not develop eczema, 10.3% in those who had undetectable OVM levels and developed eczema, and 4.2% in those who had undetectable OVM levels and did not develop eczema. In the MEE group, no infants fulfilled both risk factors, and the EA was 33.3% in infants who had detectable OVM levels and did not develop eczema, 12.5% in those who had undetectable OVM levels and developed eczema, and 2.5% in those who had undetectable OVM levels and did not develop eczema (eFigure 3 in Supplement 2).

## Discussion

This multicenter RCT with high adherence (97.6%) and follow-up rates (96.6%) determined the effect of maternal egg intake during the early neonatal period (0-5 days) on the development of EA in infants aged 12 months. The proportion of neonates aged 3 to 4 days with OVA and OVM detected in their mothers’ breast milk was higher in the MEC group than in the MEE group. However, EA at age 12 months was not significantly different between the MEC and MEE groups (9.3% vs 7.6%).

At the time of designing the current study, we hypothesized that egg consumption via breast milk in the early neonatal period prevented the development of EA, just as in the case of egg consumption in infancy.^14,15,16^ However, EA did not differ significantly between the MEC and MEE groups. There are 2 possible reasons for this. First, the intervention amount was relatively low and intervention period was relatively short. In previous trials attempting to prevent the development of EA, infants consumed gram units of egg protein for 3 to 6 months.^14,15,16,24,25,26^ However, in the current RCT, infants consumed very small amounts of egg protein (micrograms) for only 5 days. It is possible that the ingestion of larger amounts of egg protein and a longer intervention period, such as during pregnancy or after the neonatal period, might lead to different results. The second reason is the difference in the timing of egg ingestion. The gut environment, including the microbiome, changes dynamically during the neonatal period and early infancy.^42^ The current study did not evaluate the microbiome or the mother’s antibiotic use; further trials are required.

Egg allergy and sensitization to eggs tended to be higher in the MEC group, contrary to our original hypothesis. Consistently, a few studies suggested that neonatal exposure to food protein would promote the development of food allergy, unlike exposure in middle and late infancy.^29,30,31,43^ For example, in the BENEFIT (Breastfeeding and Eating Nuts and Eggs for Infant Tolerance) pilot trial, mothers were randomized to consume 6 or more eggs per week (high egg group) or 2 or fewer eggs per week (low egg group) from the neonate’s birth to age 6 months; the prevalence of EA at age 12 months was higher in the high egg group (10.0%) than in the low egg group (0.0%).^43^ Additionally, regarding milk allergy, 2 cohort trials and 1 RCT reported that early neonatal formula protein intake increased the subsequent development of milk allergy.^29,30,31^ These previous findings are similar to those observed in the present study, demonstrating that egg intake via breast milk in the early neonatal period might promote EA.

In the MEC group, the proportion of neonates with OVA and OVM detection in breast milk on days 3 and 4 was only 10.7%, which was lower than originally expected. In some previous trials, OVA was detected in mature milk in approximately 50% of infants 1 to 6 hours after their mothers ingested eggs.^32,33,34^ When designing the present study, we analyzed OVA and OVM levels in mature milk using our assay method and found that OVA and OVM were detected in 2 of the 4 cases (50%). Moreover, the detection sensitivity of OVA with our assay method was 0.20 ng/mL or greater, whereas that in previous studies was 0.57 ng/mL or greater,^32,34^ indicating our assay method is reliable. There may be differences in egg protein secretion between the colostrum and mature milk. The reason why OVA and OVM were detected in 2% of the MEE group may be due to contamination or egg consumption during pregnancy; however, in the present study, egg consumption during pregnancy has not been investigated. These issues should be assessed in future studies.

The post hoc analysis identified eczema at 1 month as a risk factor for EA development. Several cohort studies have consistently reported that eczema in early infancy is a risk factor for the subsequent development of EA.^44,45,46,47^ Furthermore, the current trial demonstrated that detecting OVM in breast milk in the early neonatal period was also a risk factor for EA. However, an Australian cohort study noted that infants with OVA detected in their mothers’ breast milk at age 3 and 6 months had a lower prevalence of EA than those without OVA detection.^33,48^ Although OVA and OVM in breast milk were not measured at birth in Australian cohort studies,^33,47^ as well as at 3 and 6 months in this trial, we hypothesized that the conflicting results might be due to a difference in the timing of exposure to egg protein. Notably, 2 RCTs on milk allergy also showed conflicting results: milk protein intake in the early neonatal period (age 0-3 days) promoted the development of milk allergy, whereas milk protein intake in early infancy (age 1-2 months) suppressed the development of milk allergy.^29,49,50^ Similarly, with regard to egg protein intake via breast milk, the effect on the subsequent development of EA may vary depending on the time of year.

### Limitations

This trial has some limitations. First, the overall prevalence of EA was 8.4%, lower than the initially expected 15%. The reason for the low prevalence of EA was speculated to be strict skin care and aggressive topical corticosteroid therapy starting at age 1 month. Aggressive topical corticosteroid therapy in infants with eczema may decrease the incidence of food allergies.^51,52^ The positive sensitization rate was 60.7%, suggesting that low sIgE levels had little clinical relevance. Therefore, the number of participants might be insufficient to examine the hypotheses of the current trial. Second, EA was diagnosed using an open OFC rather than double-blind food challenges. However, the OFC evaluators were blinded to whether infants were assigned to the MEC or MEE group. Additionally, because the OFC participants were infants, few cases presented subjective symptoms. Therefore, the effect of the open method was minimal.

## Conclusions

In this RCT, the development of EA and sensitization to egg whites was unaffected by MEC during the early neonatal period. No adverse effects were observed with either intervention.

## Notes

Supplement 1.
Trial Protocol

Supplement 2. **eFigure 1.** Intervention Procedures **eFigure 2.** Specific IgE Levels at 4 and 12 Months of Age **eFigure 3.** Prevalence of Immediate-Type Egg Allergy Based on the OVM Detection in Breast Milk on Days 3-4 and Eczema at 1 Month in Both Groups **eTable 1.** Adherence to Intervention During Admission **eTable 2.** Nutrition Methods at 1 and 4 Months of Age **eTable 3.** Nutrition Methods in Children at 7 and 10 Months of Age **eTable 4.** Proportions of Infants With OVA and OVM Detection in Breast Milk **eTable 5.** Logistic Regression Analysis of Factors Contributing to Developing IgE-Mediated Egg Allergy **eMethods.** Determination of OVA and OVM Antigen Levels in Breast Milk Using a DCP Chip Equipped With Antigen-Specific Antibodies
eReferences

Supplement 3.
Data Sharing Statement
