# Supplementary material for: Effect of Maternal Egg Intake During the Early Neonatal Period and Risk of Infant Egg Allergy at 12 Months Among Breastfeeding Mothers: A Randomized Clinical Trial
Source: JAMA Netw Open. 2023 Jul 10;6(7):e2322318. doi: 10.1001/jamanetworkopen.2023.22318 (PMC10334231; doi:10.1001/jamanetworkopen.2023.22318)
Supplement: Supplement 1. — Trial Protocol [file jamanetwopen-e2322318-s001.pdf]

National Hospital Organization Joint Clinical Research

NHO Network Collaborative Research in 2009

Task name

Evaluation of the preventive effect of early neonatal intake of hen's eggs on the development of immediate type hen's egg allergy in a randomized controlled trial: A randomized trial of postpartum egg intake for prevention of egg allergy

H29-NHO (growth) - 02

Affiliation of Principal Investigator: Clinical Research Center, National Hospital Organization Sagamihara Hospital

Principal investigator name: Sakura Sato

Version 1.0 December 11, 2017

Version 1.1 December 14, 2017

The information in this document is provided only to those directly involved in this clinical research (e.g., the head of the implementing medical institution, the administrative office of the implementing medical institution, the principal investigator, the principal investigator, clinical research collaborators, and the Ethical Review Committee). Therefore, the information cannot be disclosed to third parties unrelated to this clinical research without the prior consent of the principal investigator, except when consent is obtained from subjects participating in the clinical research.

National Hospital Organization

revision history

Version 1.0 December 11, 2017

Version 1.1 December 14, 2017

Table of contents

1. Outline 6

2. Schedule 9

3. Preface 13

3.1. Purpose 13

3.2. Background 13

3.2.1 Background on the subject 13

3.2.2. Standard treatment 13

3.2.3. Previous research 14

3.3 Benefit and risk assessment 15

4. Evaluation items 17

5. Research design 18

5.1. Design overview 18

5.2 Target number of registered cases 18

5.3. Research period 18

5.4. Scientific basis for design 19

5.5 Dosage adequacy 19

6. Participants 19

6.1. Selection criteria 19

6.2 Exclusion Criteria 20

7. Treatment 22

7.1. Test treatment 22

7.1.1 Test drug 22

7.1.2 Dosing schedule 23

7.2 Study Treatment Modification Criteria 23

7.3 Case Registration and Assignment 23

7.4. Blinding 24

7.5 Test Drug Preparation 25

7.6. Compliance with treatment methods 25

7.7 Combination therapy 25

7.8 Treatment after completion of the study 26

8. Abort criteria 27

8.1 Discontinuation of study treatment 27
8.2 Discontinuation of the study 27
8.3. Untraceable 28
9. Evaluation 29
9.1 Evaluation of Effectiveness 29
9.2 Adverse events 30
9.2.1 Definition of Adverse Event 30
9.2.2 Reporting of Serious Adverse Events 31
9.2.3. Expected adverse events 33
9.3. Safety assessment 33
9.4 Human genome and gene analysis research 33
9.5. Biomarkers 34
10. Statistics 35
10.1. Caseload design 35
10.2 Target population for analysis 35
10.3 Statistical Analysis 36
10.3.1 Efficacy Analysis 36
10.3.2 Safety Analysis 36
10.3.3. Other analysis 36
10.3.4. Intermediate analysis 37
11. Test management 37
11.1. Regulatory requirements and ethics 37
11.2 Funds and Conflicts of Interest 38
11.3. Explanation and consent 39
11.4 Protection of Research Subject Data 39
11.5 Arrangements for publicity 40
11.6 Provision of test data 40
11.7. Data quality assurance 40
11.7.1. Data management 40
11.7.2. Monitoring 40
11.7.3. Audit 41
11.7.4. Record keeping 41

11.8. Early discontinuation of the study 41
11.9 Compensation for Research Participants 42
11.9.1. Compensation for damage to health 42
11.9.2. Burden on research participants 42
11.9.3. Consultation service 42
11.10. Genome Research 42
11.11. Implementation Structure 43
11.11.1 Principal Investigator 43
11.11.2. Research Steering Committee 43
11.11.3. Research secretariat 43
11.11.4. Person responsible for statistical analysis 43
11.11.5. Data center 44
11.11.6 Data MonitoringCommittee 44
11.11.7. Effectiveness and Safety Evaluation Committee 44
11.11.8. Central inspection agency 44
11.11.9. Central decision-making body 44
11.11.10. Indemnity insurance 45
11.11.11. Planned implementing medical institution and principal investigator 45
11.11.12. Personal Information Manager 45
11.11.13. Person in charge of case registration and allocation 45
12: Literature 47
13.1 Abbreviations and terms 49
14. Annex 49
14.1 Attachment of the study drug 49
14.2. Annex 49

1. Outline

1.1. Schema

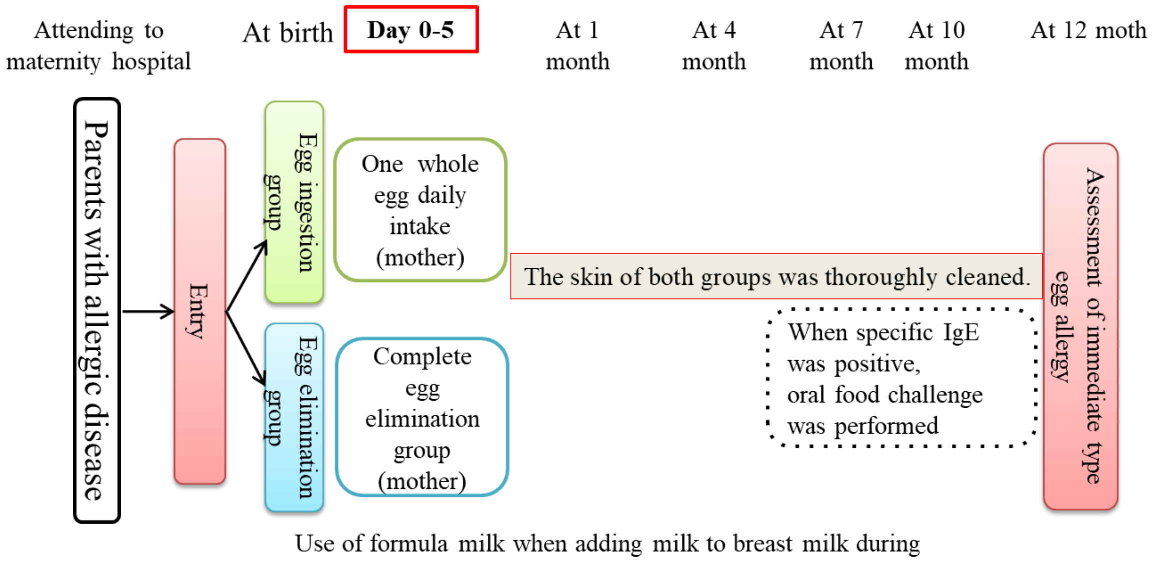

	At 1 month	At 4 month	At 7 month	At 10 month	At 12 moth
Medical examination	+	+			+
Blood test		+			+
Questionnaire distribution	+	+	+	+	
Incentives	+	+	+	+	+

1.2. Purpose

To examine whether the ingestion of hen’s eggs by the mother in the early neonatal period, before sensitization to hen’s eggs is established, is effective in preventing the development of immediate type egg allergy.

1.3. Evaluation items

purpose	evaluation item
Major	

✓ Evaluation of the preventive effect of egg protein intake via breast milk in early neonates on the development of immediate type chicken egg allergy	✓ Presence of immediate type egg allergy at 1 year of age
Secondary	
✓ Testing for sensitization to hen's eggs by early neonatal ingestion via breast milk<br>✓ Examine the impact on the development of other food allergies<br>✓ To examine the effect of daily intake of one egg by mothers immediately after delivery on the amount of egg protein in breast milk<br>✓ Assess for adverse events during the study	✓ Presence of sensitization to hen's eggs<br>✓ Sensitization and development of other food allergies<br>✓ Amount of chicken egg protein in breast milk<br>✓ Adverse Events (AEs)

1.4. Participants

Main selection criteria

1) Children at high risk of developing food allergies: One of the parents currently has an allergic disease (at least one of the following: atopic dermatitis, bronchial asthma, food allergy, allergic rhinitis, and allergic conjunctivitis).

(2) Mothers and children who provided consent to participate in the study during outpatient obstetric care at each participating facility.

Main exclusion criteria

(1) Premature baby

(2) Children with a birth weight of less than 2300 g

(3) Children with severe neonatal paralysis (Apgar score of fewer than 3 points at 5 minutes)

Children who need to be hospitalized in the NICU

(5) Children who cannot take breast milk after the age of 2 days

(6) Children for whom the attending physician has determined that the test cannot be performed.

(7) Children whose mothers are allergic to chicken eggs

1.5. Target number of cases

380 cases (egg intake group: 190 cases, egg removal group: 190 cases)

1.6. Research period

The period of registration of the research subjects: The Central Ethical Review Committee for Clinical Research of the National Hospital Organization (hereinafter referred to as the "Central Ethical Review Committee") 2 years and 6 months after approval

Observation (follow-up) period: 1 year from the last enrollment of the study subjects

Total research period: 3 years and 6 months (planned period: November 2017 to May 2021).

1.7. Research design

A multicenter, single-blinded (outcome data evaluators), randomized controlled trial

1.8. Principal Investigator

Director, Laboratory of Etiology and Pathogenesis, Clinical Research Center, Sagamihara Hospital, National Hospital Organization

Sakura Sato

Address: 18-1 Sakuradai, Minami-ku, Sagamihara City, Kanagawa Prefecture

TEL: 042-742-8311

2. Schedule

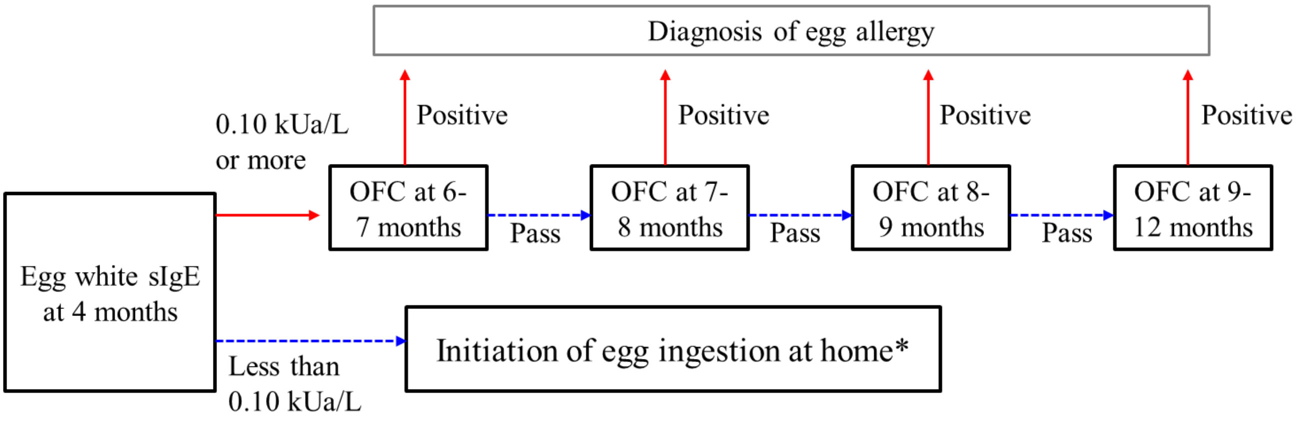

*If symptoms appear, contact to attending physician immediately.
The attending physician carefully assessed and diagnosed for egg allergy based on the blood test results at 12 months.

Prenatal

The study will be explained to the mothers attending the outpatient clinic of each participating facility, and the mothers and their children who consented to the study will be tentatively registered as the subjects of the study. Appendix 1 will be provided to the mothers in the egg removal group.

While in the hospital

After the birth of the child, it will be confirmed that the exclusion criteria are not met and the child will be enrolled in the study. The infants will be randomly divided into two groups: an early egg intake group in which the mother consumes one egg per day between 0-5 days of neonatal age, and an egg removal group in which the mother completely removes eggs from the diet (hospital food or food brought in). See Appendix 1 for information on bring-your-own food. If breast milk alone is insufficient for the infant's feeding volume, a general formula will be used.

On the fourth day of life, 1 mL of breast milk will be collected three times in total (8:30 am, 10:30 am, and 1:30 pm). The specimens will be sent to the University of Tokushima and the amount of chicken egg protein will be measured using a DLC chip.

One pre- and post-breastfeeding weighing will be performed on day 4.

After leaving the hospital

The mother is free to consume eggs at home without any specific guidance.

At 1 month of neonatal age

Adequate skin care instructions for the child's skin will be provided by Appendix 2.

A questionnaire survey (Appendix 3) will be conducted on the mothers' egg intake.

At 4 months of neonatal age

Adequate skin care instruction will be provided for the child's skin.

A questionnaire survey (Appendix 3) will be conducted on the mothers' egg intake.

The children’s blood will be tested and evaluated for the presence of egg sensitization. In cases where consent is obtained, 2 mL of whole blood will be collected for serum storage at the same time. The total volume of blood collected after obtaining consent is 5 mL.

Specimens will be stored at -20°C and collected by a specialized company for delivery to the Sagamihara Hospital Clinical Research Center.

At 5 months of neonatal age

The weaning process will be started. If the results of the child's blood test do not show sensitization to eggs (egg white specific IgE > 0.10 UA/mL), the parents will be instructed to promote the child to eat eggs at home. If sensitization is observed, the food oral stress test will be performed, as described below. If any of the test results are positive, the child will not undergo any further oral challenge tests.

At 6-7 months of age: 1/32 of a whole egg

At 7-8 months of age: 1/8 of a whole egg

At 8-9 months of age: 1/2 whole egg

At 9-12 months of age: whole roasted eggs

Immediate egg allergy will be diagnosed and assessed as the occurrence of the primary endpoint if the child has obvious immediate symptoms of egg consumption at home and is diagnosed as having immediate egg allergy on subsequent examination by an allergist and if any food oral stress test result is positive.

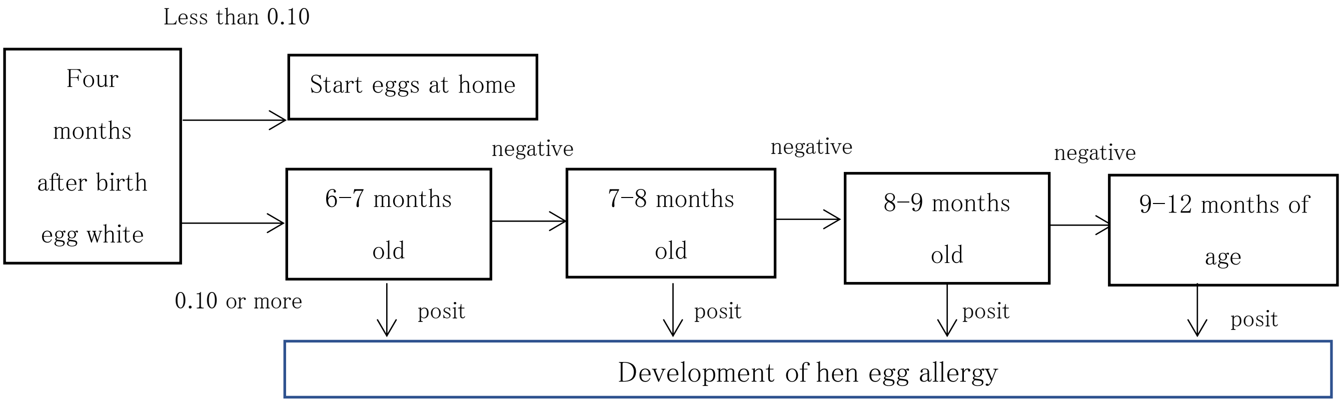

At 7 and 10 months of age

A questionnaire on egg intake of children and mothers at home (Appendix 4) will be mailed. The questionnaire will be on a postcard and sent to the research office of the Sagamihara Hospital. The postcards will be placed in an envelope and sent to the home from each research facility.

At 12 months of age

Evaluate for the presence of immediate hen's egg allergy.

The children's blood will be tested and evaluated for the presence of egg sensitization. In cases where consent is obtained, 2 mL of whole blood will be collected for serum storage at the same time. The total volume of blood collected after obtaining consent is 5 mL.

Infants who do not progress with egg consumption will be instructed to eat eggs at home if the egg white-specific IgE test result is class 0 or 1 at 12 months of age. If the egg white-

specific IgE test is class 2 or higher, the child will be evaluated at the hospital using an oral food stress test. Immediate egg allergy will be diagnosed and evaluated as the occurrence of the primary endpoint if the child has obvious and immediate symptoms when eating eggs at home and is diagnosed as having an immediate egg allergy at a subsequent examination by an allergist or if the child tests positive in any of the food oral stress tests.

Children and mothers will be asked to complete a questionnaire (Appendix 4) on their egg intake.

3. Preface

3.1. Purpose

Evaluation of the preventive effect of maternal egg intake in the early neonatal period on the development of immediate type chicken egg allergy

Evaluation of the effect of mother's egg intake on the prevention of immediate egg allergy in the early neonatal period, before sensitization to chicken eggs is established.

3.2. Background

3.2.1 Background on the subject

Prevalence of food allergy

In recent years, the prevalence of food allergies has been on the rise worldwide.[1,2] In Japan, about 10% of infants and toddlers develop food allergies, and a study of 3-year-old children reported that the prevalence rate doubled in the past 9 years, and a study of schoolchildren reported that it increased by approximately 1.7 times in the past 10 years.[3,4] Food allergies have become a social problem, requiring human and financial resources in many fields such as medicine, nutrition, and education. Egg allergy is the most common cause of immediate food allergy in Japan, accounting for 38.3% of all food allergy cases, and future countermeasures are urgently needed.[5]

3.2.2. Standard treatment

Preventing the development of food allergies

With regard to prevention, in 2000, the American Academy of Pediatrics announced that children at high risk of developing allergies should not be given eggs until the age of 2 years, but in 2008, it retracted this statement, stating that there is no clear evidence for delaying the

start of weaning to prevent the onset of food allergy.[6] Subsequent studies have suggested the possibility of preventing the onset of food allergy through early feeding,[7,8] but there is currently no established method of prevention, including for high-risk children with a family history of allergic disease.[7,8] There are also no intervention studies on nursing mothers that have examined the effects of allergy prevention.

Dealing with Food Allergies

The principle of the management of children with food allergies is to wait for the acquisition of tolerance (a state in which symptoms no longer occur even when ingested) while removing the causative food.[9] Oral stress tests should be conducted periodically, and follow-up should be conducted while determining whether tolerance has been acquired. However, children with a history of anaphylaxis are less likely to acquire tolerance,[10] and the QOL of affected children and their families is greatly reduced.

3.2.3. Previous research

The necessity of primary prevention against immediate hen egg allergy

Prevention of the onset of food allergy is a major issue in the treatment of food allergies. The prevention of onset can be divided into three stages: primary prevention (prevention of sensitization itself), secondary prevention (prevention of onset in sensitized children), and tertiary prevention (early acquisition of tolerance in affected children).

Previous studies have focused on chicken egg allergy, which is the most frequent type of allergy. Regarding secondary prevention, it was reported that the incidence of egg allergy after the age of 1 year was significantly lower in the early intake group of fully breastfed 3-month-old infants who received eggs until the age of 6 months compared with the full breastfeeding group who received only breast milk.[7] In a study of 6-month-old children with

eczema, the incidence of egg allergy at 1 year of age was significantly lower in the early egg intake group than in the egg removal group.[8] The above study suggested the possibility of secondary prevention by early egg intake in infancy.[9] As for tertiary prevention, we found that infants with immediate type egg allergy acquired tolerance to egg white by ingesting trace amounts of egg white.[11] However, early neonatal intervention for primary prevention has not been investigated so far.

Time of sensitization in food allergy

Many infants test positive for egg white-specific IgE antibodies at 3 months of age,[12] but the above intervention study excluded children who had already been sensitized. Although the timing of sensitization is unclear, exposure to dietary proteins during the early neonatal period may be associated with subsequent sensitization. Sensitization

It is thought that some type of intervention before the onset of action may lead to primary prevention. However, there is a concern that administering chicken eggs to newborn infants, whose digestive tract is still immature, may place a heavy physiological burden on the digestive tract. Therefore, we hypothesized that the administration of eggs via breastfeeding during the early neonatal period (0-5 days) might have a primary preventive effect on egg sensitization. As a general practice in Japan, it is acceptable for mothers to consume one egg per day or remove eggs from their diet. In addition, the intervention period was limited to only 5 days, so the burden was minimal.

3.3 Benefit and risk assessment

In recent years, adverse effects of egg consumption, such as hypercholesterolemia, have been ruled out, and in 2016, it was reported that consumption of one egg per day reduced the risk of stroke and did not increase the risk of coronary artery disease.[13] There are no specific

issues with mothers consuming one egg per day and avoiding eggs during normal breastfeeding and parenting practices. In this study, the duration of egg intake or removal was limited to 5 days, and the burden was low if the intake was restricted to eggs only.

Blood tests will be performed at 4 and 12 months, but only for children at high risk of developing food allergies. The results of these tests will be used to guide weaning food intake. Although this may increase the frequency of testing, it also provides a benefit to the affected child in terms of a more detailed assessment of allergy.

For the food oral stress test, the target dose will be set at a small amount equivalent to 1/32 of a whole egg, and if the test result is negative, the target dose will be increased gradually to 1/8 of a whole egg, 1/2 of a whole egg, and a whole egg; if the test result is positive, the dose will not be increased any further. We have earlier reported that such a method of conducting food oral stress tests in small amounts and by stages is safer than the conventional method of food oral stress tests.[14] Although there is a risk of symptom induction during the food oral stress test, unnecessary elimination without a correct diagnosis can be avoided.

4. Evaluation items

purpose	evaluation item
Major<br>✓ Evaluation of the preventive effect of egg protein intake via breast milk in early neonates on the development of immediate type chicken egg allergy	✓ Presence of immediate type egg allergy at 1 year of age
Secondary	
✓ Testing for sensitization to hen's eggs by early neonatal ingestion via breast milk<br>✓ Examine the impact on the development of other food allergies<br>✓ To examine the effect of daily intake of one egg by mothers immediately after delivery on the amount of egg protein in breast milk<br>✓ Assess for adverse events during the study	✓ Presence of sensitization to hen's eggs<br>✓ Sensitization and development of other food allergies<br>✓ Amount of chicken egg protein in breast milk<br>✓ Adverse Events (AEs)

The presence or absence of immediate type allergy will be evaluated by interviews and food oral stress tests, and the presence or absence of sensitization will be evaluated using specific IgE antibody titers. The definitions and measurement methods are described in Section 9. Evaluation."

5. Research design

5.1. Design overview

Multicenter open-label randomized controlled trial: In addition to the five facilities of the National Hospital Organization, three facilities, including an obstetric hospital, will be added as cooperating facilities of Sagamihara Hospital, for a total of eight facilities participating in the study.

Blocking: Yes (considering the facility)

5.2 Target number of registered cases

Overall, 380 cases (egg intake group 190 cases, egg removal group 190 cases)

5.3. Research period

The study will begin with the approval of the Central Ethics Review Committee and end when the last study participant completes the last observation date, when the study is discontinued, or when the study participant stops visiting the hospital (i.e., when the principal investigator or sub-investigator can no longer contact the patient).

The research period for each participant shall be from the date of obtaining consent to the date of the last observation or the date of the decision to discontinue the research. The date of the decision to discontinue is the date on which the principal investigator or sub-investigator decides to discontinue the research with the research participant.

Planned enrollment period: 2 years and 6 months after approval by the Central Ethics Review Committee

Planned observation period: 1 year from enrollment of the last study participant

Planned total research period: 3 years and 6 months

5.4. Scientific basis for design

When examining the effect of early intervention in preventing the onset of food allergies, evaluation is difficult because of recall bias in backward-looking questionnaires. In addition, because the risk of developing egg allergy varies with family history, number of cousins, method of delivery, and history of eczema, it is difficult to evaluate patients without randomization because background factors may be biased.

The target population is children and mothers of children at high risk of developing food allergies.

We believe that the intervention will be needed only for the first 4 or 5 days of life and that there will be no major nutritional problems.

5.5 Dosage adequacy

We believe that consuming one hen's egg per day is within the range of amounts consumed in typical daily life and is not a problem.

Even in the removal group, eggs will be removed from the diet only for 4 or 5 days, and we believe that there will be no nutritional problems.

6. Participants

6.1. Selection criteria

The child and mother shall meet all of the following criteria

(1) Target

Children and mothers at high risk of developing food allergies: Children[15,16] and mothers of children with at least one of the two parents having a current allergic disease (at least one of the following: atopic dermatitis, bronchial asthma, food allergy, allergic rhinitis (perennial or seasonal), allergic rhinitis).

(2) Mothers and children who were informed about the study during obstetric care and provided consent to participate in the study.

The basis for selection criteria:

1) Because it is considered most useful for screening children at high risk for food allergy and this criterion has been used in various allergy prevention studies[15,16]

(2) Ethical Considerations

Provisional registration of children and mothers of children who meet the selection criteria will be performed before birth.

6.2 Exclusion Criteria

Children who fall into any of the following categories shall be excluded from this study:

(1) Children under 37 weeks of age

(2) Children with a birth weight of less than 2300 g

(3) Children with severe neonatal paralysis (Apgar score of fewer than 3 points at 5 minutes)

4) Children who need to be admitted to the NICU

(5) Children who are expected to be unable to consume breast milk at all after two age of 2 days.

(6) Children for whom the physician-in-charge has determined that it is not feasible to conduct this study.

(7) Children whose mothers are allergic to eggs and cannot consume them

The rationale for setting exclusion criteria:

(1)-(5) Because it strongly affects the primary endpoint

(6)–(7) Because it is impossible to continue the research.

Children and mothers of children confirmed to not meet the above exclusion criteria after birth will be enrolled in the study.

7. Treatment

Investigational treatment is defined as an experimental treatment, marketed drug, placebo, or medical device intended to be administered to a research participant in accordance with the research protocol.

7.1. Test treatment

7.1.1 Test drug

In the egg intake group, the mother will be provided with and consume one cooked egg (boiled egg) per day from days 0 to 5. In the egg-removed group, the mother will be provided with a meal from which the eggs have been removed.

If blood test results do not show sensitization to eggs (egg white specific IgE 0.10 UA/mL or higher) after 4 months of age, the child will be weaned on eggs at home from 5 months of age, and the weaning food will be aimed at half a whole egg at home. If sensitization is observed, an oral food challenge test using cooked whole egg powder with a target dose of 1/32 whole eggs will be performed at 6-7 months of age. If the test result is negative, an oral challenge test with a target dose of 1/8 whole egg at 7-8 months and 1/2 whole egg at 8-9 months will be performed using cooked whole egg powder. If both test results are negative, a food challenge test will be performed at 9-12 months with a target dose of whole poached eggs.

ny food oral stress test result is positive.

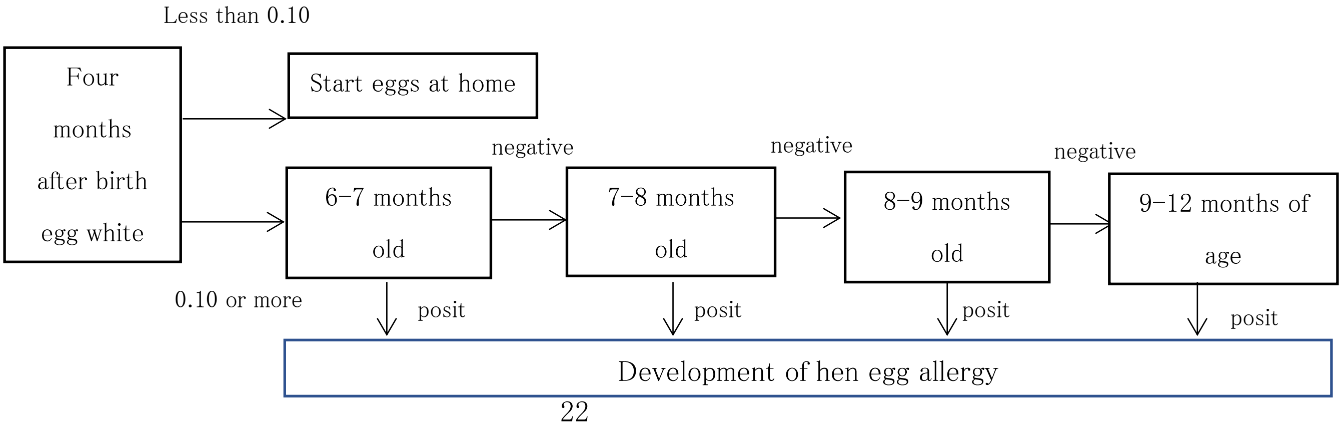

7.1.2 Dosing schedule

The day of birth of the infant to be studied (mother's day of delivery) will be set as day 0, and the mothers in the egg intake group will consume one additional cooked chicken egg per day, in addition to their normal diet, from day 0 to day 5. The mothers in the egg-removal group will receive a hospital diet that eliminates eggs, including processed foods, from days 0 to 5. If the discharge date is after day 6, the intervention will be up to day 5, and the diet after day 6 will include regular food intake. If the discharge date is day four, the intervention will be up to day age 4 and the data will be entered to that effect.

There are no dietary restrictions after discharge.

7.2 Study Treatment Modification Criteria

No changes will be made before day 0-5 days of age.

7.3 Case Registration and Assignment

(1) The person in charge of case enrollment at the research secretariat will randomize each facility and prepare an allocation table for the research ID (unique ID for this study) and the intervention group (egg intake group or removal group) for each facility. The allocation list for each facility will be maintained by the research secretariat until the end of the study. The forms will be placed with the study ID and intervention group in a sealed envelope, glued together, and with only the study ID written on the envelope cover. The envelopes will be packed and sent in advance to each research facility.

The researcher in charge of registration at each facility will fill out the provisional registration form with the research ID, allergy information of the parents (especially that the mother is not allergic to eggs), and the birth order of the child, and fax it to the research secretariat when the research consent is obtained. The research ID will be assigned to each facility in the order of provisional registration. The provisional registration of the case is

complete when the fax is sent to the research secretariat. After provisional registration is complete, the envelope with the corresponding research ID on the cover will be handed over to the other subcontractor in charge of allocation (nurse, midwife, etc.). The researcher in charge of the allocation will open the envelope, sign the envelope, and inform the nutrition department of each facility of the dietary requirements of the egg intake group or the egg removal group. The mothers of the egg-removed group will be given a printout (Appendix 1) of the precautions for bringing in food. The research secretariat will collect the envelopes later and confirm their signatures.

(iii) After the birth of the child, it will be confirmed that the exclusion criteria are not met, and the child will be enrolled in the study. If the exclusion criteria are met, the child will be excluded.

Research subcontractors at each hospital will maintain a table of correspondence between the study participant’s personal information and research IDs, and only research IDs will be used when registering with central monitoring.

The strata of the allocation adjustment factors will be randomized using the substitution block method, as per the facility. Randomization will be performed blindly by using the envelope method.

Random allocation method: stratified substitution block method, the envelope method

Allocation stratification factor: per facility

7.4. Blinding

This is an open-label study and no blinding procedures will be applied.

7.5 Test Drug Preparation

The eggs consumed by the mothers will be boiled and prepared by the nutritional department of each facility.

When a hen egg dietary oral challenge test is required, a dietary oral challenge test with graded target doses (1/32 whole eggs, 1/8 whole eggs, and 1/2 whole eggs) will be conducted using standardized cooked whole egg powder.

In the food oral stress test with a target dose of 1/32 whole eggs, heated whole egg powder manufactured by the Kewpie Corporation Laboratory will be used. The hygienic safety of hen’s egg powder will be confirmed by microbiological examination and the amount of antigen confirmed by ELISA to be equivalent to that of 1/32 M-sized heated hen’s egg per 2 g package.

For the food oral challenge test with target doses of 1/8 whole eggs and 1/2 whole eggs, heated whole egg powder manufactured by Nippon Ham will be used. Four packets are equivalent to half of an entire egg. All powders will be stored at room temperature.

We have previously reported on the safety and efficacy of these food-oral challenge tests.[17]

7.6. Compliance with treatment methods

We believe that the intervention will be easy to implement because it will take place during the 5 days of hospitalization immediately after the neonatal birth. Participants will be asked to ensure that they consume the meals allocated to them by the hospital’s feeding service.

7.7 Combination therapy

From birth 0-5 days, the egg intake group will consume one cooked chicken egg per day, and the egg removal group will avoid eggs.

When eczema appears, skincare guidance will be provided, and for poor improvement, topical group 4 steroids will be used.

Children who do not show egg sensitization at 4 months will be encouraged to eat eggs at home from 5 to 6 months.

Children with egg sensitization at 4 months of age will be given a graded food oral challenge test starting with 1/32 whole eggs at 6-7 months. (See section 7.1.2, Dosing schedule)

7.8 Treatment after completion of the study

The examination itself is within the scope of normal practice, and the patient will return to normal practice after the examination.

8. Abort criteria

8.1 Discontinuation of study treatment

The study treatment of a research participant will be discontinued if any of the following occur:

If the mother of the child develops an allergy to hen’s eggs

If the mother of the child is unable to eat after delivery

If the research participant or his/her caretaker requests that the study treatment be discontinued

If the research participant's study is discontinued

If the principal investigator or sub-investigator determines that it is appropriate to discontinue the study treatment for any other reason.

Patients who discontinue study treatment will continue to be observed during the study period.

8.2 Discontinuation of the study

A research participant's study will be discontinued if any of the following occur:

Death of a research participant

The research participant becomes untraceable due to relocation, etc.

Request for withdrawal of consent by a substitute

The case is found to be ineligible after registration

Any other violation of the research protocol is found.

The study is discontinued at the relevant medical institution

The entire study is discontinued

The principal investigator or sub-investigator determines that it is appropriate to discontinue the study for any other reason.

Research participants and substitutes may withdraw consent, that is, withdraw from a clinical trial at any time at their request, and a research participant's clinical trial may be terminated at any time for reasons related to safety, behavior, or management, as determined by the principal investigator or sub-investigator.

Data collected before the withdrawal of consent may continue to be used.

If the research participant and the substitute wish to discontinue the study, the research participant may request that specimens that have been collected but not tested be destroyed, and the principal investigator must maintain a record of this.

8.3. Untraceable

If a research participant does not make a scheduled medical visit and cannot be contacted by the clinical trial site, they will be considered untraceable.

If a research participant does not show up on the required visit date, the following measures will be taken:

・The implementing medical institution will attempt to contact the research participant, coordinate a return visit as soon as possible, advise them on the importance of adhering to the designated visit, and confirm whether they are willing to continue with the clinical trial.

If the research participant appears to be untraceable, every effort will be made by the principal investigator, sub-investigator, or their representative to re-establish contact with them. Attempts to make contacts will be documented.

Notwithstanding these attempts, if the research participant cannot be contacted, the clinical trial will be deemed to have been terminated and the primary reason will be untraceable.

9. Evaluation

9.1 Evaluation of Effectiveness

Primary endpoint

Observations: Presence of immediate hen's egg allergy at 1 year of age

Method of investigation: The physician in charge diagnoses the patient based on the results of episodes or food oral stress tests. The food oral stress test is performed at different target doses (1/32 whole egg, 1/8 whole egg, 1/2 whole egg, and whole egg). The food oral stress test is conducted using standardized heated whole egg powder (1/32 whole egg oral stress test is conducted by Kewpie Corporation Laboratory, and 1/8 whole egg and 1/2 whole egg oral stress tests are conducted using heated whole egg powder manufactured by Japan Ham).
Immediate egg allergy is diagnosed when the egg white specific IgE level is 0.10 UA/mL or higher and the patient has obvious immediate symptoms after egg ingestion at home and is diagnosed as having immediate egg allergy at a subsequent consultation with an allergist, or when any food oral stress test result is positive, and the primary endpoint of incidence and evaluation is reached.
Immediate-type symptoms are defined in accordance with the anaphylaxis guidelines established by the Japanese Society of Allergy.[14,18] Skin symptoms due to contact alone are not considered immediate symptoms and should be excluded.

Secondary endpoints

(1) Blood test results

Observations: Total IgE antibody titer, egg white/ovomucoid-specific IgE antibody titer, milk/casein, and wheat/ω5 gliadin-specific IgE antibody titers

Methods: Blood tests performed at 4 and 12 months of age to measure eosinophil count, TARC, total IgE, egg white-specific IgE, ovomucoid-specific IgE, milk-specific IgE, wheat-specific IgE, and ω5 gliadin-specific IgE using the ImmunoCAP method.

A specific IgE antibody titer of 0.10 UA/mL or higher is considered sensitization.

Blood samples will be taken within one month before and after the specified period.

(2) Egg protein content in breast milk

Observation: Amount of chicken egg protein in breast milk

Research method: On the fourth day after birth, 1 mL of breast milk is collected three times in total (1, 3, and 6 h after egg intake). The specimens will be sent to the University of Tokushima for measurements using a DLC chip.

(3) Clinical assessment

Observation: Presence of milk or wheat allergy

Method of investigation: The physician in charge diagnoses the patient based on the results of episodes or food oral stress test results.

Background factor

Observation items: (1) allergic diseases in the family, number of compatriots, (2) cesarean delivery, birth week, birth weight, sex, (3) breast milk intake during hospitalization, and (4) breast milk intake after discharge, intake, and frequency of maternal chicken egg intake.

Survey method: 1) Questionnaire survey of the mothers' class, 2) Forward-looking survey of medical records, 3) Weight measurement before and after breastfeeding, and 4) Questionnaire survey

9.2 Adverse events

9.2.1 Definition of Adverse Event

Adverse events

All unfavorable or unintended injuries or illnesses or signs thereof (including abnormal clinical laboratory values) occurring in a research participant, whether or not causally related to the research conducted. means.

Serious adverse events

An adverse event is defined as any of the following

1) Those that lead to death

2) Life-threatening

3) Those requiring hospitalization or extended hospitalization for treatment (emergency hospitalization for treatment of adverse events, excluding hospitalization scheduled in advance)

4) Permanent or significant disability or dysfunction.

5) Congenital anomalies in the offspring

6) Any other event deemed to be a medically significant condition (a significant medical event that, although not immediately life-threatening or resulting in death or hospitalization, is likely to endanger the patient or require treatment or therapy to avoid the consequences listed in the definition above).

9.2.2 Reporting of Serious Adverse Events

All serious adverse events from the start of the study treatment to the date of the last observation or discontinuation will be collected.

After the initial serious adverse event report, the principal investigator or sub-investigator will follow the event until it is confirmed (death, recovery, or loss to follow-up). The reporting procedure is as follows:

(1) In the event of an adverse event that is the subject of an emergency report, the principal investigator or sub-investigator at the institution where the adverse event occurred will immediately take appropriate measures and record the event in the medical records.

(2) Serve to submit a written SAE report to the principal investigator and research steering committee members within 24 h of learning the occurrence of the event.

(3) If necessary, the principal investigator and the research steering committee members will inquire about the suspicious matter to the principal investigator and sub-investigators at the institution where the adverse event occurred and confirm that it is a serious adverse event.

(4) The principal investigator and members of the research steering committee shall prepare an SAE statement after examining the urgency, importance, and degree of impact of the contents. Depending on the content of the SAE, enrollment may be suspended or terminated, or the research protocol and consent documents may be revised with the approval of the principal investigator.

(5) The principal investigator and members of the research steering committee will finalize the SAE report, and if the report is confirmed to be a serious adverse event, will disseminate the contents of the SAE report and the status and results of the response to all institutions. This will be done as much as possible within 72 h of submission of the SAE report to the principal investigator.

(6) The principal investigators of the research facilities where the adverse events occurred report the confirmed adverse events to the head of the relevant medical institutions, the principal investigators, and the Central Ethics Review Committee in the form "Form⑳. Serious Adverse Event Report (201504)" to the head of the relevant medical institution, principal investigator, and central ethics review committee. This will be carried out as much as possible within 72 h after the finalization of the SAE opinion letter.

(7) In the event of an unpredictable serious adverse event that is causally related to the research, the principal investigator shall report to the Minister of Health, Labor, and Welfare in collaboration with the head of the institution where the adverse event occurred, using the form provided in the "Guidance on Ethical Guidelines for Medical Research Involving Human Subjects," and shall also disclose the status of the response and the results on the principal investigator institution; the status and results of the response shall be published on the website of the principal investigator institution.

(8) If new information becomes available after reporting an adverse event that is the subject of an emergency report, the principal investigator shall make an additional report and repeat (1) through (8).

9.2.3. Expected adverse events

Adverse reactions may be induced when the child ingests the chicken eggs.

We have already reported on the safety of ingesting chicken egg powder in oral loading studies.

9.3. Safety assessment

The principal investigator or sub-investigator will evaluate the adverse event in the research participant and, if signs (including laboratory values) and symptoms are part of the diagnosis, record the name of the diagnosis rather than the individual signs and symptoms in the case report whenever possible.

This study itself is within the normal scope of practice and is not considered a safety issue.

9.4 Human genome and gene analysis research

This study does not evaluate the genetic evaluation items subject to the "Ethical Guidelines for Human Genome/Gene Analysis Research."

9.5. Biomarkers

In this study, total IgE, egg white-specific IgE, ovomucoid-specific IgE, milk-specific IgE, wheat-specific IgE, and ω5 gliadin-specific IgE levels will be measured using the ImmunoCAP method. In cases where consent has been obtained, the serum will be stored and may be used when a new hypothesis arises in the future. In such cases, we will submit a new research plan and obtain approval from the Ethics Committee before use. The serum will be collected at each research facility and stored at -20°C. Delivery will be outsourced to a specialized company to collect them from each facility and store them at the Sagamihara Hospital Clinical Research Center. The storage period shall be 10 years. When the tests are outsourced, they will be disposed of immediately after the measurement. Disposals will be carried out in accordance with hospital regulations.

10. Statistics

10.1. Caseload design

In Japan, food allergies occur in approximately 20% of infants.[19] The most common causative food of food allergy is hen's egg.[5] In this study, only children at high risk of developing food allergy will be included, and the incidence of egg allergy in the egg removal group is assumed to be 15%. The prevalence of egg allergies in the egg-removed group is assumed to be 15%, and the preventive effect of early intervention is assumed to be 60%. Assuming a dropout rate of 20%, the total number of cases is 350. The total number of patients required is 380, considering the differences between the centers.

An interim analysis will be performed for each of the 100 enrolled patients, and entry will be discontinued when the Peto cutoff criterion of 0.001 is reached. In the final analysis, a P value of less than 0.05. will be used as the significance level.

The number of deliveries per year at the participating centers will range from 200 to 2000, for a total of 4000 deliveries per year at the eight centers. Approximately 50% of the children have allergic diseases in either parent, and the number of children to be studied will be approximately 2000 per year. Assuming that 10% of these children will participate in the study, this can be achieved in 2 years.

10.2 Target population for analysis

For all efficacy assessments, the analysis in the largest eligible population for analysis (FAS) will be the primary analysis, and the analysis in the eligible population conforming to the study protocol (PPS) will be conducted as a reference. For safety analysis, an analysis of the target population for safety analysis will be conducted. The target population is defined as follows:

Largest Full Analysis Set (FAS)

All enrolled patients will comprise the largest population for analysis (FAS). However, cases with serious violations of the study protocol (failure to obtain consent or serious violations of study procedures) will be excluded.

The target population conforming to the research protocol (PPS: Per Protocol Set)

The population shall exclude cases with violation of eligibility and exclusion criteria from the FAS to the provisions of the study protocol, including study treatment and combination therapy. Cases with erroneous food provision during the first 5 days after delivery shall also be excluded.

Safety analysis population

A population of all enrolled patients, excluding those who did not receive any study treatment.

10.3 Statistical Analysis

Professor Mitsuyoshi Urashima, Laboratory of Molecular Epidemiology, The Jikei University School of Medicine, who is a research collaborator, assist in the preparation of the research protocol and be responsible for the statistical analysis in this study.

10.3.1 Efficacy Analysis

This will be conducted on the largest analysis set (FAS: full analysis set).

10.3.2 Safety Analysis

All safety analyses will be performed on the safety evaluation population.

10.3.3. Other analysis

Not implemented

10.3.4. Intermediate analysis

It will be carried out by the Data Monitoring Committee.

An interim analysis will be performed for each of the 100 enrolled patients, and entry will be discontinued when the cutoff criterion of Peto is below 0.001.

11. Test management

11.1. Regulatory requirements and ethics

This study will be conducted in accordance with the study protocol and the following

World Medical Association Declaration of Helsinki

Ethical Guidelines for Medical Research Involving Human Subjects

Act on the Protection of Personal Information

After approval by the Central Ethical Review Committee, permission from the director of each implementing medical institution is required for the implementation of the relevant research, preparation and revision of the research protocol, and changes to the principal investigators.

Principal investigators are responsible for the following

The progress of the research and the occurrence of adverse events associated with the implementation of the research will be reported once a year. When the research is completed or terminated at the affiliated institution, a report to that effect will be submitted to the head of the affiliated institution for deliberation by the Central Ethics Review Committee.

Serious adverse events or other serious safety findings that are required by the Central Ethics Review Committee procedures should be reported to the head of the institution to which the researcher belongs for discussion by the Central Ethics Review Committee.

If the researcher obtains facts or information that impair or may impair the ethical validity or scientific rationality of the research and that may affect the continuation of the research, the researcher shall report to the head of the institution to which the researcher belongs.

When facts or information that undermine or may undermine the appropriateness of the conduct of research or the reliability of research results are obtained, they are reported to the head of the institution to which the researcher belongs.

The study was conducted at the institution in accordance with the study protocol and all applicable regulatory requirements.

11.2 Funds and Conflicts of Interest

This study is funded by the "Research Grant-in-Aid for Operation of the National Hospital Organization."

The cost of eggs provided for meals, breast milk collection and testing, mailing of questionnaires, egg powder used in the stress test, and quo cards will be paid from the research funds. The costs of blood tests (eosinophil count, TARC, egg white, and ovomucoid-specific IgE antibody titer, milk, casein, and wheat, and ω5 gliadin-specific IgE antibody titer) will be covered by insurance.

There are no financial interests or conflicts of interest to be described in this study. The management of the personal conflicts of interest of the investigators will be in accordance with the regulations of each institution.

11.3. Explanation and consent

The principal investigator or sub-investigator gives the mother of the subject child an explanatory document that meets the requirements set by the regulatory requirements described in Section 11.1. Regulatory requirements and ethics, " which was approved by the Central Ethical Review Committee for Clinical Research, and explains the research to her. After giving the mother sufficient time to think about the study and confirming that she has a good understanding of the details of the study, the mother is asked to participate in the study. If the mother agrees to participate in the study, she will be required to sign the consent form. The principal investigator or sub-investigator confirms that the consent form includes the name of the researcher who explained it, the date of the explanation, the name of the patient who received the explanation, and the date of consent. A copy of the consent form will be handed to the mother, and the original will be maintained in the medical record or a storage place designated by the medical institution.

If the explanatory document is revised, the revised document will be provided to the research participant, the revision will be explained, and the research participant's intention to continue participation in the research will be confirmed. If a research participant who has agreed to participate in the research expresses their intention to withdraw their consent in a written or oral form, the principal investigator or sub-investigator will take the necessary measures according to the content of the withdrawal.

11.4 Protection of Research Subject Data

Due consideration will be given to the protection of privacy in accordance with the Personal Information Protection Law. The participants' clinical data will be managed using an anonymized research ID. The results of the study will be presented at conferences and

published in papers; however, sufficient care will be taken to ensure that individuals cannot be identified. This research will be conducted with the permission of the Central Ethics Committee, and Chizuko Sugisaki, a research collaborator, will be assigned as a personal information manager.

11.5 Arrangements for publicity

The results of this study will be published within 2 years of the completion of the study by a presentation at a conference or in a paper. The data collected during this study belong to the National Hospital Organization (NHO), and prior consent of the principal investigator (PI) is required to publish or present the results of the study in any publication or article abstract.

11.6 Provision of test data

The outline of this study will be registered in the clinical trial registration system (UMIN-CTR) of the University Hospital Medical Information Network Research Center and will be updated as appropriate according to changes in the research protocol and the progress of the study.

If the obtained samples and information are to be provided to outside parties after the completion of this study, it is necessary to obtain approval from the Central Ethical Review Committee for the research plan, in which new samples and information will be used.

11.7. Data quality assurance

11.7.1. Data management

The participants' clinical data will be managed using a consolidated and anonymized study ID, with personal information removed.

11.7.2. Monitoring

Monitoring will be enforced periodically by the Data Monitoring Committee.

Recruitment status, randomization, adherence to the intervention, follow-up status, outcome, and adverse events

Monitoring of the input data in which questions have arisen shall be conducted as needed. It is responsible for making appropriate inquiries.

Entry will be discontinued when the cutoff value of Peto falls below 0.001.

Periodic monitoring reports on the study’s progress will be conducted annually.

11.7.3. Audit

The Department of Clinical Research, National Hospital Organization Mie Hospital (Laboratory of Allergy Disease Treatment and Development, Mizuo Nagao) will be in charge of the audit.

11.7.4. Record keeping

The original materials, etc., will be kept at the implementing medical institution, and the collected information, etc. will be kept at the principal investigator's facility for at least 5 years after the completion of the research. When destroying records, consideration should be given to protecting the privacy of the research subjects.

11.8. Early discontinuation of the study

The principal investigator may discontinue the research if the balance of risks and benefits across the research participants is unacceptable, including when recommendations are made by the Central Ethics Review Board or other authorities. They may also discontinue study inclusion or the inclusion of specific sites when the number of study participants who discontinued due to noncompliance with the study protocol, regulatory requirements, problems with implementation procedures, or administrative reasons is high.

11.9 Compensation for Research Participants

11.9.1. Compensation for damage to health

This research will be covered by the clinical research compensation insurance. If any health damage occurs to the research participant owing to the implementation of this research, each implementing medical institution will be responsible for the treatment. The costs of the treatment of health damage will be covered by the health insurance of the research participant and his/her mother. In the unlikely event that unexpected serious health damage occurs and a causal relationship to this research is established, compensation may be paid according to the extent of the damage.

11.9.2. Burden on research participants

This study will be conducted entirely within the scope of insurance, and the research participants will be responsible for any out-of-pocket medical expenses. Participation in the study will not result in any increase in costs compared with routine medical care.

The research participants who participate in this study will be paid 1,000 yen on Quo cards as rewards during outpatient visits and questionnaire surveys (five times in total).

11.9.3. Consultation service

The point of contact for consultation from research participants and their related persons shall be the principal investigator, and the contact information will be included in the explanatory document.

11.10. Genome Research

This research is not subject to the "Ethical Guidelines for Human Genome and Gene Analysis Research."

11.11. Implementation Structure

11.11.1 Principal Investigator

Sakura Sato, Clinical Research Center, Sagamihara Hospital, National Hospital Organization

18-1 Sakuradai, Minami-ku, Sagamihara-shi, Kanagawa 252-0392

TEL:042-742-8311 FAX:042-742-5314 Email: s-satou@sagamihara-hosp.gr.jp

Duties: Final approval of the research protocol and overseeing the entire research through the Research Steering Committee.

11.11.2. Research Steering Committee

Clinical Research Center, Sagamihara Hospital, National Hospital Organization

18-1 Sakuradai, Minami-ku, Sagamihara-shi, Kanagawa 252-0392

TEL:042-742-8311 FAX:042-742-5314 Email: s-satou@sagamihara-hosp.gr.jp

Duties: To conceive and plan this study and review the entire study. Safety information management.

11.11.3. Research secretariat

Ken-ichi Nagakura, Department of Pediatrics, National Hospital Organization Sagamihara Hospital

18-1 Sakuradai, Minami-ku, Sagamihara-shi, Kanagawa 252-0392

TEL: 042-742-8311 FAX: 042-742-5314 Email: k-nagakura@sagamihara-hosp.gr.jp

Duties: Progress management, coordination, and record-keeping of this research as a whole under the direction of the Research Steering Committee.

11.11.4. Person responsible for statistical analysis

Mitsuyoshi Urashima, Laboratory of Molecular Epidemiology, The Jikei University School of Medicine, Tokyo, Japan

Duties: To perform the statistical analysis in this study. The data will be fixed by the research data manager at each stage when every 100 cases are reached, and when the target number of cases is reached, the data will be sent to the person responsible for statistical analysis.

11.11.5. Data center

Clinical Research Center, Sagamihara Hospital, National Hospital Organization

Duties: Case registration, data management, and central monitoring in this study will be performed.

11.11.6 Data MonitoringCommittee

Makoto Suzuki, Director, Nago Clinic

Morimitsu Tomikawa, Director, Odasaga Pediatric Allergy Clinic

Clinical Research Center, Sagamihara Hospital, National Hospital Organization (Laboratory of Allergic Diseases: Chizuko Sugisaki)

Duties: To monitor the implementation of this research using e-mail and other means.

11.11.7. Effectiveness and Safety Evaluation Committee

Clinical Research Center, Sagamihara Hospital, National Hospital Organization (Laboratory of Diagnostic and Therapeutic Development: Tomoma Fukutomi)

Duties: To review and make recommendations regarding efficacy and safety in response to consultation with the principal investigator.

11.11.8. Central inspection agency

Hiroshi Kido, Division of Pathophysiology and Metabolism of Biodefense and Infectious Diseases, Research Center for Enzymology of Diseases, The University of Tokushima

Duties: To perform central testing for food antigens in breast milk.

11.11.9. Central decision-making body

Department of Pediatrics, National Hospital Organization Sagamihara Hospital

Duties: To make a central judgment of the test results.

11.11.10. Indemnity insurance

Ken-ichi Nagakura, Department of Pediatrics, National Hospital Organization Sagamihara Hospital

Duties: To be in charge of liability and indemnity insurance for this study. We will join Mitsui Sumitomo Insurance Co.

11.11.11. Planned implementing medical institution and principal investigator

Sakura Sato, National Hospital Organization Sagamihara Hospital

Masaki Futamura, National Hospital Organization Nagoya Medical Center

Hideo Kaneko, National Hospital Organization Nagara Medical Center

Toshinori Kobayashi, National Hospital Organization Yokohama Medical Center

Isamu Kamimaki, National Hospital Organization Saitama Hospital

Michimasa Fujiwara, National Hospital Organization Fukuyama Medical Center

Hiroshi Koga, National Hospital Organization Beppu Medical Center

Eiju Fujita, Aiwa Hospital

Hiroaki Taniguchi, Rokko Island Konan Hospital

Eiji Makita, Saitama Medical Center, Jichi Medical University

11.11.12. Personal Information Manager

Chizuko Sugisaki, Clinical Research Center, Sagamihara Hospital, National Hospital Organization

Duties: To carry out information management.

11.11.13. Person in charge of case registration and allocation

Fumiko Goto, Clinical Research Center, Sagamihara Hospital, National Hospital Organization

Duties: Responsible for case registration and allocation. This person will not provide explanations, consent, or evaluation to the research participants.

12: Literature

1. Skripak JM, Matsui EC, Mudd K, Wood RA. Natural history of IgE-mediated cow milk allergy. *J Allergy Clin Immunol*. 2007;120(5):1172-1177. doi: 10.1016/j.jaci.2007.08.023.

2. Sicherer SH, Muñoz-Furlong A, Godbold JH and Sampson HA. US prevalence of self-reported peanut, tree nut, and sesame allergy:11-year follow-up. *J Allergy Clin Immunol*. 2010;125(6):1322-1326. doi: 10.1016/j.jaci.2010.03.029.

3. Bureau of Social Welfare and Public Health, Tokyo Metropolitan Government. All Metropolitan Survey of 3-Year-Old Children regarding Allergic Diseases; 2009.

4. *Report on the 2013 Survey Project on Health Management in School Life: Japan School Health Association*.

5. Akiyama H, Imai T, Ebisawa M. Japan food allergen labeling regulation–history and evaluation. *Adv Food Nutr Res*. 2011;62:139-171. doi: 10.1016/B978-0-12-385989-1.00004-1.

6. Greer FR, Sicherer SH, Burks AW, American Academy of Pediatrics Committee on Nutrition, American Academy of Pediatrics Section on Allergy and Immunology. Effects of early nutritional interventions on the development of atopic disease in infants and children: The role of maternal dietary restriction, breastfeeding, timing of introduction of complementary foods, and hydrolyzed formulas. *Pediatrics*. 2008;121(1):183-191. doi: 10.1542/peds.2007-3022.

7. Du Toit G, Roberts G, Sayre PH, Bahnson HT, Radulovic S, Santos AF, et al. *N Engl J Med*. 2016;372:803-813. doi: 10.1056/NEJMoa1414850.

8. Natsume O, Kabashima S, Nakazato J, et al.. Two-step egg introduction for prevention of egg allergy in high-risk infants with eczema (PETIT): A randomised, double-blind, placebo-controlled trial. *Lancet*. 2017;389(10066):276-286. doi: 10.1016/S0140-6736(16)31418-0.

9. Ebisawa M. Management of food allergy in Japan "food allergy management guideline 2008 (revision from 2005)" and "guidelines for the treatment of allergic diseases in schools". *Allergol Int*. 2009;58(4):475-483. doi: 10.2332/allergolint.09-RAI-0143.

10. Ohtani K, Sato S, Syukuya A, et al.. Natural history of immediate-type hen's egg allergy in Japanese children. *Allergol Int*. 2016;65(2):153-157. doi: 10.1016/j.alit.2015.10.005.

11. Okada, Y., Yanagida, N., Sato, S. Ebisawa M. WAO J. 2016;9:31. https://doi.org/10.1186/s40413-016-0121-4

12. Kaori I, et al. *Allergy*. 2006;55:140-150.

13. Alexander DD, Miller PE, Vargas AJ, Weed DL, Cohen SS. Meta-analysis of Egg Consumption and Risk of Coronary Heart Disease and Stroke. *J Am Coll Nutr*. 2016;35(8):704-716. doi: 10.1080/07315724.2016.1152928.

14. Yanagida N, Okada Y, Sato S, Ebisawa M. New approach for food allergy management using low-dose oral food challenges and low-dose oral immunotherapies. *Allergol Int*. 2016;65(2):135-140. doi: 10.1016/j.alit.2015.10.010.

15. Boyce JA, Assa'ad A, Burks AW, et al.. Guidelines for the Diagnosis and Management of Food Allergy in the United States: Summary of the NIAID-Sponsored Expert Panel Report. *J Allergy Clin Immunol*. 2010;126(6):1105-1118. doi: 10.1016/j.jaci.2010.10.008.

16. Muraro A, et al. *Allergy*. 2015;69:590-601.

17. Yanagida N, Sato S, Ebisawa M. Pediatric allergy. *Arerugi*. 2016;65(3):193-199. doi: 10.15036/arerugi.65.193.

18. Ebisawa M, Ito K, Fujisawa T, Committee for Japanese Pediatric Guideline for Food Allergy, The Japanese Society of Pediatric Allergy and Clinical Immunology, The Japanese Society of Allergology. Japanese guidelines for food allergy 2017. *Allergol Int*. 2017;66(2):248-264. doi: 10.1016/j.alit.2017.02.001.

19. Report on the Tokyo metropolitan institute of health and safety research in 2014;13**.**

appendix.

13.1 Abbreviations and terms

AE: adverse event, adverse event

14. Annex

14.1 Attachment of the study drug

There are no special drugs used in the test.

14.2. Annex

Attachment 1: Materials to be given to those in the egg removal group

Attachment 2: Flowchart on Skin Care

Appendix 3: Questionnaire at 1 and 4 months

Appendix 4: Questionnaires at 7, 10, and 12 months

Statistical analysis plan

Evaluation of the preventive effect of early neonatal intake of hen's eggs on the development of immediate type hen's egg allergy in a randomized controlled trial: A randomized trial of postpartum egg intake for prevention of egg allergy

1. Purpose of the Study

To examine whether the ingestion of hen's eggs by the mother in the early neonatal period, before sensitization to hen's eggs is established, is effective in preventing the development of immediate type egg allergy.

1.1. Primary endpoints

Evaluation: Percentage of presence of immediate type egg allergy at 1 year of age

1.2. Secondary endpoints

Presence of sensitization to hen's eggs, cow's milk and wheat

Sensitization and development of cow's milk and wheat allergies

Amount of hen's egg protein in breast milk

2. General matters in statistical analysis

2.1. Intermediate analysis

An interim analysis will be performed for each of the 100 enrolled patients, and entry will be discontinued when the cutoff criterion of Peto is below 0.001.

2.2. Monitoring

Ensure that the study is conducted in compliance with the protocol, that data are collected accurately, and that informed consent is properly obtained.

2.3 Data Handling

The data will be based on data that has been fixed after the study has been completed.

For the test values, each detection limit is assigned for data above or below the detection limit.

2.4 Handling of Missing Values

The subject who is absent due to discontinuation or other reasons shall be evaluated as absent for that period of time.

2.5. significance and confidence levels

The significance level is 5% two-sided, and the confidence interval is 95% two-sided.

3. Population to be analyzed

3.1. The population to be analyzed

Largest analysis set (FAS: Full Analysis Set)

All enrolled cases will be considered the largest population for analysis (FAS). However, cases of serious violations of the study protocol (failure to obtain consent, serious violations of study procedures) will be excluded.

Per Protocol Set (PPS): A population of subjects that conforms to the research protocol.

The population will exclude from the FAS those cases in which there were violations of eligibility/exclusion criteria or violations of concomitant use of prohibited drugs or concomitant use of prohibited therapies against the provisions of the research protocol, such as study treatment or concomitant therapy.

Safety Analysis Population

The population of all enrolled patients, excluding those who did not receive any study treatment at all.

3.2. Correspondence with statistical analysis items

For all efficacy evaluations, the analysis in the largest analysis population (FAS) willbe the primary analysis, with an analysis in the population of subjects (PPS) that conforms to the study protocol as a reference. The safety analysis will be performed in the safety analysis population.

4. Analysis plan for data

Risk ratios, 95% confidence intervals, and p-values will be analyzed. The presence or absence of immediate egg allergy, cow's milk or wheat allergy will be analyzed using the Fisher's exact test. Blood test data and egg protein levels in breast milk will be analyzed using the Wilcoxon rank-sum test.

Statistical significance was set at $P < 0.05$.
