# Supplementary material for: Effect of Maternal Egg Intake During the Early Neonatal Period and Risk of Infant Egg Allergy at 12 Months Among Breastfeeding Mothers: A Randomized Clinical Trial
Source: JAMA Netw Open. 2023 Jul 10;6(7):e2322318. doi: 10.1001/jamanetworkopen.2023.22318 (PMC10334231; doi:10.1001/jamanetworkopen.2023.22318)
Supplement: Supplement 2. — eFigure 1. Intervention Procedures eFigure 2. Specific IgE Levels at 4 and 12 Months of Age eFigure 3. Prevalence of Immediate-Type Egg Allergy Based on the OVM Detection in Breast Milk on Days 3-4 and Eczema at 1 Month in Both Groups eTable 1. Adherence to Intervention During Admission eTable 2. Nutrition Methods at 1 and 4 Months of Age eTable 3. Nutrition Methods in Children at 7 and 10 Months of Age eTable 4. Proportions of Infants With OVA and OVM Detection in Breast Milk eTable 5. Logistic Regression Analysis of Factors Contributing to Developing IgE-Mediated Egg Allergy eMethods. Determination of OVA and OVM Antigen Levels in Breast Milk Using a DCP Chip Equipped With Antigen-Specific Antibodies eReferences [file jamanetwopen-e2322318-s002.pdf]

Supplementary Online Content

Nagakura KI, Sato S, Shinahara W, et al. Effect of maternal egg intake during the early neonatal period and risk of infant egg allergy at 12 months among breastfeeding mothers: a randomized clinical trial. *JAMA Netw Open*. 2023;6(7):e2322318. doi:10.1001/jamanetworkopen.2023.22318

eFigure 1. Intervention Procedures

eFigure 2. Specific IgE Levels at 4 and 12 Months of Age

eFigure 3. Prevalence of Immediate-Type Egg Allergy Based on the OVM Detection in Breast Milk on Days 3-4 and Eczema at 1 Month in Both Groups

eTable 1. Adherence to Intervention During Admission

eTable 2. Nutrition Methods at 1 and 4 Months of Age

eTable 3. Nutrition Methods in Children at 7 and 10 Months of Age

eTable 4. Proportions of Infants With OVA and OVM Detection in Breast Milk

eTable 5. Logistic Regression Analysis of Factors Contributing to Developing IgE-Mediated Egg Allergy

eMethods. Determination of OVA and OVM Antigen Levels in Breast Milk Using a DCP Chip Equipped With Antigen-Specific Antibodies

eReferences

This supplementary material has been provided by the authors to give readers additional information about their work.

© 2023 Nagakura KI et al. *JAMA Network Open.*

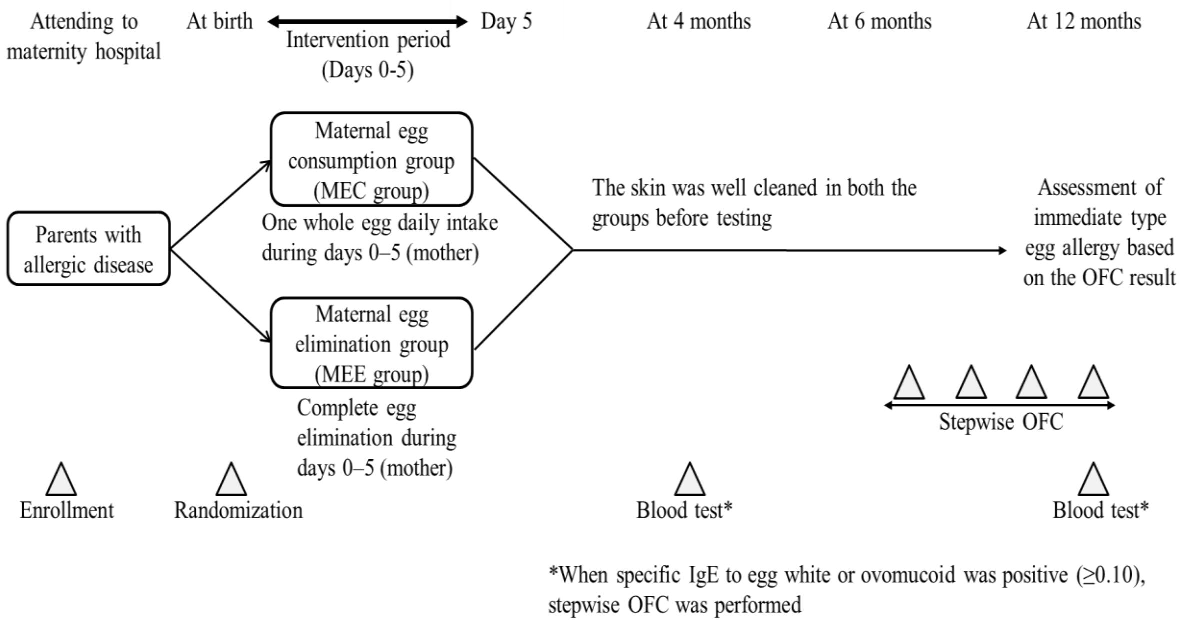

eFigure 1. Intervention procedures. OFC: oral food challenge.

© 2023 Nagakura KI et al. *JAMA Network Open.*

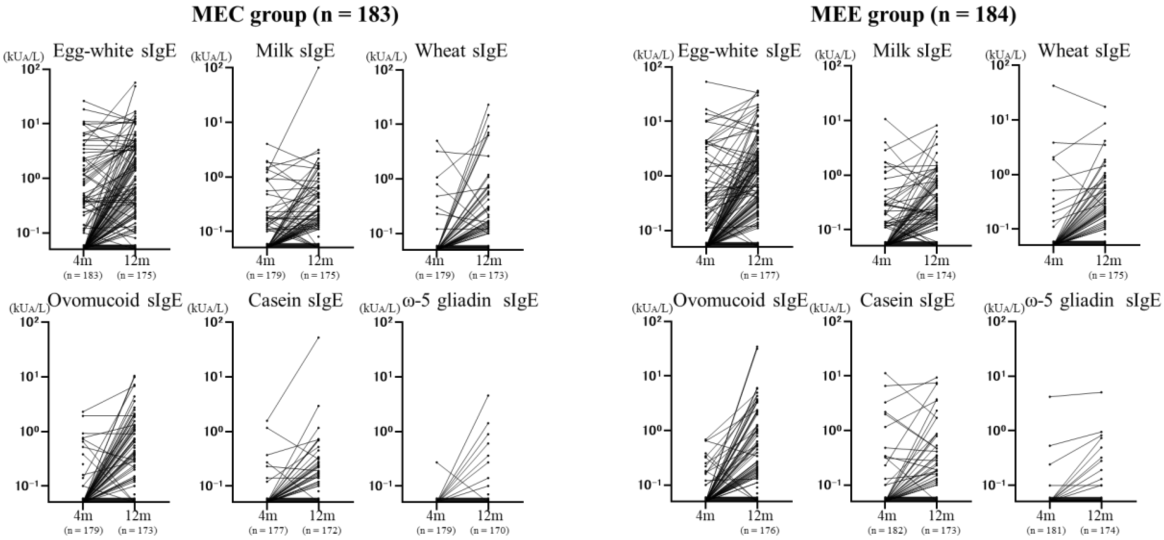

eFigure 2. Specific IgE levels at 4 and 12 months of age.
At 4 months of age, sensitizations to egg white, OVM, milk, casein, wheat, and ω-5 gliadin were respectively observed in 30.1%, 7.1%, 16.4%, 3.8%, 4.4%, and 0.5% of participants in the MEC group and 26.6%, 8.7%, 18.5%, 8.2%, 6.0%, and 1.6% of participants in the MEE group. At 12 months of age, sensitizations to egg white, OVM, milk, casein, wheat, and ω-5 gliadin were respectively observed in 62.8%, 31.1%, 37.2%, 18.6%, 25.7%, and 4.4% of participants in the MEC group and 58.7%, 33.2%, 39.7%, 20.1%, 25.0%, and 5.4% of participants in the MEE group.

© 2023 Nagakura KI et al. *JAMA Network Open.*

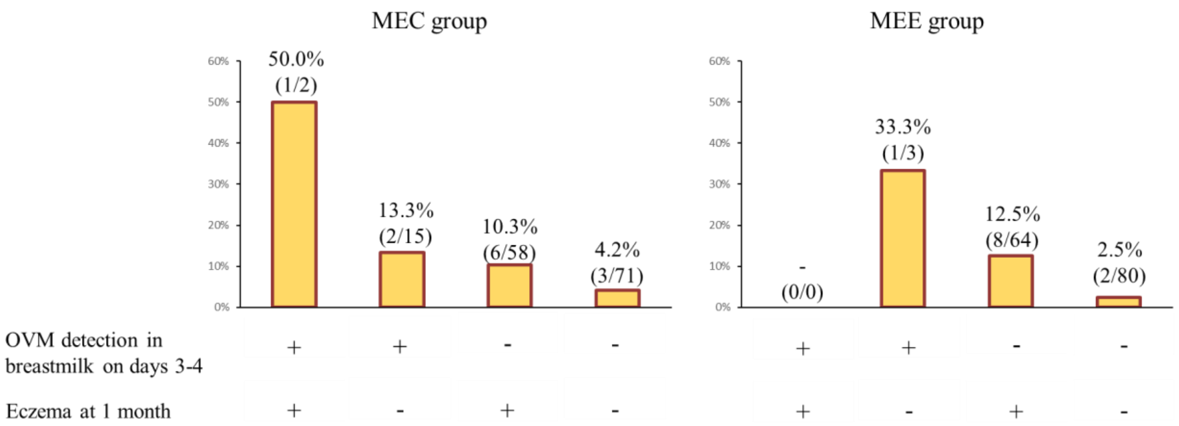

eFigure 3. Prevalence of immediate-type egg allergy based on the OVM detection in breast milk on days 3-4 and eczema at 1 month in both groups.

© 2023 Nagakura KI et al. *JAMA Network Open.*

eTable 1. Adherence to intervention during admission.

		MEC group (n = 190)	MEE group (n = 190)
Mothers	Mothers who ingested a whole hen's egg	187 (98.4%)	-
	Mothers who completely eliminated hen's eggs	-	184 (96.8%)
Infants	Amount of consumption of breastmilk per feeding on day 3, median (IQR)	12 ml (4-28ml)	12 ml (4-31ml)

To determine the amount of consumption of breastmilk per feeding on day 3, we measured the weight of the infants before and after each feeding.

© 2023 Nagakura KI et al. *JAMA Network Open.*

eTable 2. Nutrition methods at 1 and 4 months of age.

		1 month of age<br>MEC group (n = 183)	MEE group (n = 184)	*P* value	4 months of age<br>MEC group (n = 183)	MEE group (n = 184)	*P* value
Infants	Feeding	Exclusive breast milk: 57 (31.1%)<br>Mixed: 117 (63.9%)<br>Formula feeding: 2 (1.1%)<br>Unknown: 6 (3.3%)	Exclusive breast milk: 57 (31.0%)<br>Mixed: 119 (64.7%)<br>Formula feeding: 0 (0.0%)<br>Unknown: 8 (4.3%)	>.99	Exclusive breast milk: 88 (48.1%)<br>Mixed: 74 (40.4%)<br>Formula feeding: 11 (6.0%)<br>Unknown: 10 (5.5%)	Exclusive breast milk: 85 (46.2%)<br>Mixed: 81 (44.0%)<br>Formula feeding: 11 (6.0%)<br>Unknown: 7 (3.8%)	>.99
Mothers	Number of egg consumption	≥2 times/day: 22 (12.0%)<br>1 time/day: 47 (25.7%)<br>4-6 times/week: 65 (35.5%)<br>2-3 times/week: 39 (21.3%)<br>1 time/week: 4 (2.2%)<br>0 time: 0 (0.0%)<br>Unknown: 6 (3.3%)	≥2 times/day: 16 (8.7%)<br>1 time/day: 70 (38.0%)<br>4-6 times/week: 42 (22.8%)<br>2-3 times/week: 41 (22.3%)<br>1 time/week: 7 (3.8%)<br>0 time: 1 (0.5%)<br>Unknown: 7 (3.8%)	>.99	≥ 2 times/day: 18 (9.8%)<br>1 time/day: 58 (31.7%)<br>4-6 times/week: 47 (25.7%)<br>2-3 times/week: 46 (25.1%)<br>1 time/week: 4 (2.2%)<br>0 time: 0 (0.0%)<br>Unknown: 10 (5.5%)	≥ 2 times/day: 18 (9.8%)<br>1 time/day: 54 (29.3%)<br>4-6 times/week: 54 (29.3%)<br>2-3 times/week: 44 (23.9%)<br>1 time/week: 6 (3.3%)<br>0 time: 1 (0.5%)<br>Unknown: 7 (3.8%)	>.99
	Amount of egg consumption	≥3: 1 (0.5%)<br>2: 14 (7.7%)<br>1: 131 (71.6%)<br>1/2: 26 (14.2%)<br>1/4: 5 (2.7%)<br>Unknown: 6 (3.3%)	≥3: 0 (0.0%)<br>2: 6 (3.3%)<br>1: 133 (72.3%)<br>1/2: 27 (14.7%)<br><1/4: 11 (6.0%)<br>Unknown: 7 (3.8%)	>.99	≥3: 1 (0.5%)<br>2: 18 (9.8%)<br>1: 123 (67.2%)<br>1/2: 27 (14.8%)<br>≤1/4: 4 (2.2%)<br>Unknown: 10 (5.5%)	≥3: 0 (0.0%)<br>2: 21 (11.4%)<br>1: 112 (60.9%)<br>1/2: 29 (15.8%)<br><1/4: 15 (8.2%)<br>Unknown: 7 (3.8%)	>.99

© 2023 Nagakura KI et al. *JAMA Network Open.*

eTable 3. Nutrition methods in children at 7 and 10 months of age.

		7 months of age			10 months of age		
		MEC group (n = 175)	MEE group (n = 180)	*P* value	MEC group (n = 175)	MEE group (n = 173)	*P* value
Breast milk		151 (86.3%)	156 (86.7%)	>.99	131 (74.9%)	124 (71.7%)	.55
Solid food	Times per day	3 times: 3 (1.7%), 2 times: 102 (58.3%), 1 time: 67 (38.3%), None: 3 (1.7%)	3 times: 6 (3.3%), 2 times: 110 (61.1%), 1 time: 62 (34.4%), None: 2 (1.1%)	>.99	3 times: 156 (89.1%), 2 times: 19 (10.9%), 1 time: 0 (0.0%), None: 0 (0.0%)	3 times: 144 (83.2%), 2 times: 27 (15.6%), 1 time: 1 (0.6%), None: 1 (0.6%)	>.99
	Egg yolk	77 (44.0%)	73 (40.6%)	.52	128 (73.1%)	129 (74.6%)	.81
	Egg white	30 (17.1%)	29 (16.1%)	.89	127 (72.6%)	136 (78.6%)	.21
	Cow's milk	41 (23.4%)	40 (22.2%)	.80	121 (69.1%)	119 (68.8%)	>.99
	Wheat	95 (54.3%)	90 (50.0%)	.46	169 (96.6%)	162 (93.6%)	.22
	Soy	125 (71.4%)	116 (64.4%)	.17	171 (97.7%)	163 (94.2%)	.11
	Fish	136 (77.7%)	128 (71.1%)	.18	173 (98.9%)	171 (98.8%)	>.99
	Chicken	47 (26.9%)	40 (22.2%)	.33	171 (97.7%)	166 (96.0%)	.38
	Rice	171 (97.7%)	174 (96.7%)	.75	175 (100.0%)	172 (99.4%)	.50
	Peanut	1 (0.6%)	2 (1.1%)	>.99	9 (5.1%)	10 (5.8%)	.82

© 2023 Nagakura KI et al. *JAMA Network Open.*

eTable 4. Proportions of infants with OVA and OVM detection in breast milk.

		MEC group (n = 150)	MEE group (n = 147)	RR (95%CI)
Days 3-4	OVA detection	16 (10.7%)	3 (2.0%)	5.23 (1.56-17.56)
	OVM detection	17 (11.3%)	3 (2.0%)	5.55 (1.66-18.55)
1 month	OVA detection	8/148 (5.4%)	10/145 (6.9%)	0.78 (0.32-1.93)
	OVM detection	6/148 (4.1%)	6/145 (4.1%)	0.98 (0.32-2.97)

OVA: ovalbumin; OVM: ovomucoid; MEC: maternal egg consumption; MEE: maternal egg elimination; RR: risk ratio; CI: confidence interval.

© 2023 Nagakura KI et al. *JAMA Network Open.*

eTable 5. Logistic regression analysis of factors contributing to developing IgE-mediated egg allergy

	Adjusted RRs (95%CI)	*P* value
Randomization to the MEC or MEE group	0.94 (0.46-1.92)	.86
Mother with allergic disease	2.23 (0.58-8.61)	.24
Cesarean delivery	1.63 (0.77-3.44)	.20
OVM detection in breastmilk on days 3-4	4.04 (1.58-10.32)	.003
Eczema at 1 month	2.85 (1.39-5.85)	.004

Multivariate logistic regression analysis was performed using the factors presented in this table as covariates.
RR: risk ratio, MEC: maternal egg consumption; MEE: maternal egg elimination, OVM: ovomucoid.

© 2023 Nagakura KI et al. *JAMA Network Open.*

eMethods Determination of OVA and OVM antigen levels in breast milk using a DCP chip equipped with antigen-specific antibodies

Method for quantification of OVA and OVM antigens

In this measurement system, allergenic food antigen-specific antibodies were mounted on a chip substrate and detected by sandwich ELISA. Anti-OVA and anti-OVM polyclonal antibodies were produced using Complete Freund's Adjuvant (DIFCO ADJUVANT COMPLETE FREUND, Becton, Dickinson and Company) in rabbits (Japanese white breed) at the Institute for Advanced Enzyme Research, University of Tokushima (1), and antigen affinity purification was performed to obtain anti-OVA and anti-OVM specific rabbit polyclonal IgG antibodies. Each polyclonal purified antibody was coated on a Densely Carboxylated Protein (DCP) chip by a method as described by Suzuki et al. (2) and reacted with samples containing antigen, and antigen levels were then measured by sandwich ELISA using fluorescence-labeled purified secondary antibodies. The fluorescence intensity was determined with a scanner, and the amount of antigen in the sample was calculated from the fluorescence intensity based on a previously prepared calibration curve. The CV values of spot-to-value and array-to-array differences of the prepared DCP antibody-coated chips were less than 15%.

Preparation of calibration curves for the detection of OVA and OVM antigens

Calibration curves for OVA and OVM antigens used in the antigen quantification chip were prepared as follows. Known concentrations of purified OVA (Albumin from chicken egg white, Sigma-Aldrich) or purified OVM antigens (Trypsin inhibitor from chicken egg white, Type Ⅲ-O, Sigma-Aldrich) were added to chips coated with purified anti-OVA or anti-OVM rabbit polyclonal IgG antibodies. The measurable range of OVA antigen was 0.20–25 ng/mL, and that of OVM antigen was 0.78–25 ng/mL.

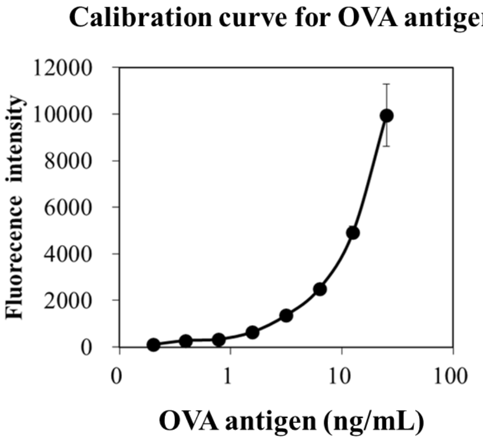

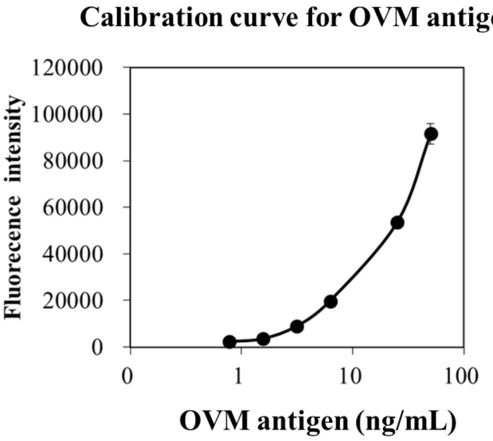

A calibration curve was prepared with the amount of antigen as the horizontal axis and the fluorescence intensity as the vertical axis. The amount of antigen in the sample was calculated from the fluorescence intensity.

To create a calibration curve under measurement conditions similar to breast milk, Morinaga E-milk (for 0–1-year-old babies) (MORINAGA MILK INDUSTRY CO., LTD.), for which the OVA and OVM antigens in the powdered milk solution were previously confirmed to be below the detection limit, was centrifuged (eFig 5) and used as antigen lysate and Blank solution for the calibration curve creation.

© 2023 Nagakura KI et al. *JAMA Network Open.*

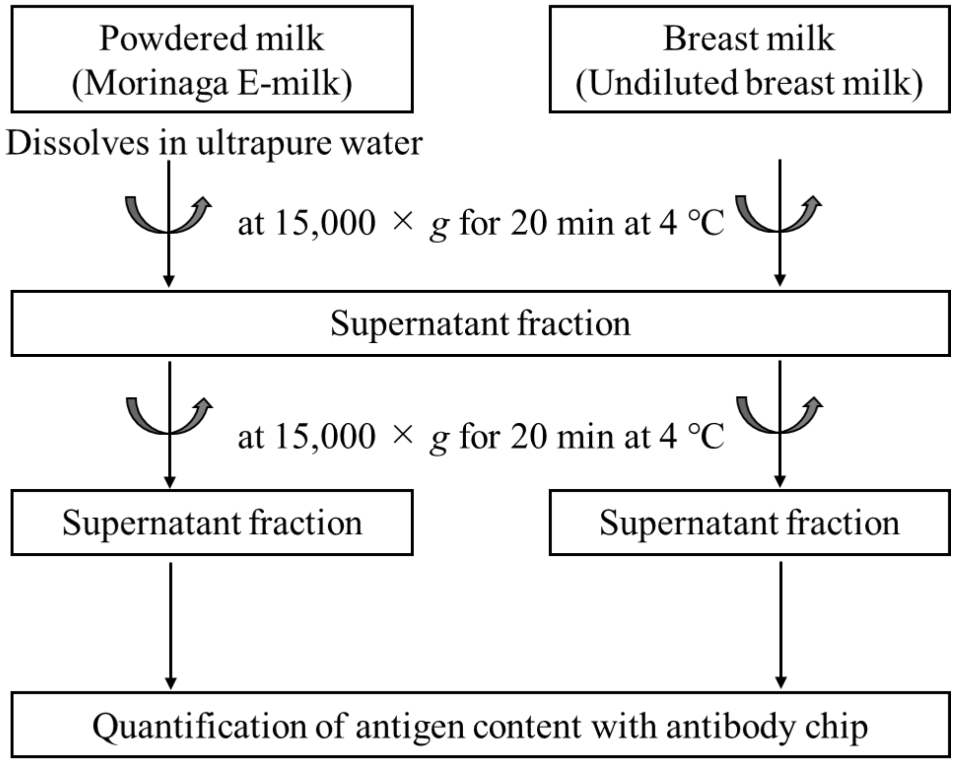

Methods for measuring OVA and OVM antigens in breast milk

The chips coated with anti-OVA or anti-OVM rabbit polyclonal IgG antibodies were reacted with centrifuged breast milk as a sample in the same way as the preparation of powdered milk antigen lysate, and the amount of OVA and OVM antigens contained was measured.

eReferences

1. Kido H, Izumi K, Otsuka H, Fukusen N, Kato Y, Katunuma N. A chymotrypsin-type serine protease in rat basophilic leukemia cells: evidence for its immunologic identity with atypical mast cell protease. *J Immunol*. 1986;136(3):1061-1065.
2. Suzuki K, Hiyoshi M, Tada H, et al. Allergen diagnosis microarray with high-density immobilization capacity using diamond-like carbon-coated chips for profiling allergen-specific IgE and other immunoglobulins. *Anal Chim Acta*. 2011;706(2):321-327.

© 2023 Nagakura KI et al. *JAMA Network Open.*
