# Supplementary material for: Effect of Maternal Egg Intake During the Early Neonatal Period and Risk of Infant Egg Allergy at 12 Months Among Breastfeeding Mothers: A Randomized Clinical Trial
Source: JAMA Netw Open. 2023 Jul 10;6(7):e2322318. doi: 10.1001/jamanetworkopen.2023.22318 (PMC10334231; doi:10.1001/jamanetworkopen.2023.22318)
Supplement: Supplement 3. — Data Sharing Statement [file jamanetwopen-e2322318-s003.pdf]

Data Sharing Statement

Nagakura. Effect of Maternal Egg Intake During the Early Neonatal Period and Risk of Infant Egg Allergy at 12 Months Among Breastfeeding Mothers. *JAMA Netw Open*. Published July 10, 2023. doi:10.1001/jamanetworkopen.2023.22318

Data
Data available: Yes
Data types: Participant data with identifiers
How to access data: https://center6.umin.ac.jp/cgi-open-bin/ctr_e/ctr_view.cgi?recptno=R000031625
When available: With publication

Supporting Documents
Document types: None

Additional Information
Who can access the data: Noriyuki Yanagida: yana@foodallergy.jp
Types of analyses: Who can access the data: researchers whose proposed use of the data Types of analyses: for a specified purpose
Mechanisms of data availability: Mechanisms of data availability: after approval of a proposal
Any additional restrictions: Any additional restrictions: not particular for now
